# Crosstalk between noncoding RNAs and ferroptosis: new dawn for overcoming cancer progression

**DOI:** 10.1038/s41419-020-02772-8

**Published:** 2020-07-24

**Authors:** Xuefei Zhang, Lingling Wang, Haixia Li, Lei Zhang, Xiulan Zheng, Wen Cheng

**Affiliations:** Department of Ultrasonography, Harbin Medical University Cancer Hospital, 150 Haping Road, 150040 Harbin, China

**Keywords:** Cancer prevention, Cancer therapy

## Abstract

Cancer progression including proliferation, metastasis, and chemoresistance has become a serious hindrance to cancer therapy. This phenomenon mainly derives from the innate insensitive or acquired resistance of cancer cells to apoptosis. Ferroptosis is a newly discovered mechanism of programmed cell death characterized by peroxidation of the lipid membrane induced by reactive oxygen species. Ferroptosis has been confirmed to eliminate cancer cells in an apoptosis-independent manner, however, the specific regulatory mechanism of ferroptosis is still unknown. The use of ferroptosis for overcoming cancer progression is limited. Noncoding RNAs have been found to play an important roles in cancer. They regulate gene expression to affect biological processes of cancer cells such as proliferation, cell cycle, and cell death. Thus far, the functions of ncRNAs in ferroptosis of cancer cells have been examined, and the specific mechanisms by which noncoding RNAs regulate ferroptosis have been partially discovered. However, there is no summary of ferroptosis associated noncoding RNAs and their functions in different cancer types. In this review, we discuss the roles of ferroptosis-associated noncoding RNAs in detail. Moreover, future work regarding the interaction between noncoding RNAs and ferroptosis is proposed, the possible obstacles are predicted and associated solutions are put forward. This review will deepen our understanding of the relationship between noncoding RNAs and ferroptosis, and provide new insights in targeting noncoding RNAs in ferroptosis associated therapeutic strategies.

## Facts

- Resistance to apoptosis has become the main obstacle for overcoming cancer progression.
- Ferroptosis is a type of cell death characterized by excess reactive oxygen species and intracellular iron, and is totally different from apoptosis.
- NcRNAs serve as important roles in biological processes of cancer.
- Regulation of ncRNAs to ferroptosis has been partially discovered.

## Open Questions

- Can ferroptosis become the direction around which to design cancer therapy in future?
- What are the roles of ncRNAs in regulation of ferroptosis?
- Can ncRNAs become markers to filter cancer patients who are fit for ferroptosis therapy or therapeutic targets of ferroptosis inducers?

## Introduction

Cancer progression including proliferation, metastasis and chemoresistance to drugs, has become serious obstacles in cancer therapy^1^. Although multiple therapeutic manners including operation, targeted therapy, chemotherapy, and radiotherapy have shown satisfactory performance, progression occurs since cancer cells dysregulate apoptosis pathways via various manners^2,3^. Therefore, new types of cancer therapy or drugs that eliminate cancer cells are urgently needed.

Ferroptosis is a type of programmed cell death discovered in 2012^4^. Unlike apoptosis, ferroptosis is characterized by excess reactive oxygen species (ROS) and intracellular iron^5^. Superabundant ROS induces peroxidation and disintegration of lipid membrane and cell death^6^. Regulation of ferroptosis mainly depends on neutral reaction between reduced glutathione (GSH) and ROS^7^. The exchange of glutamate and cystine is mediated by systemXc^−^, which is composed of solute carrier family 7 member 11 (SLC7A11) and solute carrier family 3 member 2 (SLC3A2), and offers the substrate cystine for GSH synthesis^8,9^. Glutathione peroxidase 4 (GPX4) catelyzes interaction between GSH and ROS to reduce intracellular oxidative stress^10^. Ferroptosis inducers can be divided into two classes based on regulation of neutral reaction to ROS. Class I ferroptosis inducers such as sorafenib, erastin and sulfasalazine, serve as blockers of systemXc^−^ and result in a drop of GSH levels^11,12^. Class II ferroptosis inducers such as RSL3, FIN56, and ML162, inhibit function of GPX4^13,14^. Numerous studies have confirmed that ferroptosis inducers such as RSL3 and sorafenib eliminates cancer cells^15,16^. In addition, induction of ferroptosis via erastin and sulfasalazine improved effect of cytarabine and doxorubicin, and overcame cisplatin resistance of head and neck cancer^17,18^. This suggests that ferroptosis may become a new mechanism around which to design cancer therapy. However, use of ferroptosis in cancer therapy still faces obstacles. First, the specific mechanisms underlying ferroptosis and the interaction between ferroptosis and other processes, such as apoptosis, necrosis, and autophagy are not totally known, so how to control ferroptosis in cancer is in dark. Second, ferroptosis occurs in normal cells. Ferroptosis has been shown to induce the elimination of nerve cells in Parkinson’s disease^19^. In addition, in acute kidney injury, ferroptosis participated in the death of renal tubular epithelial cells^20^. Therefore, use of ferroptosis inducers may generate complications. New regulatory factors should be recognized to understand the true appearance of ferroptosis in cancer.

Noncoding RNAs (ncRNAs) are RNAs that account for nearly 98% of transcriptome^21^. According to length and shapes, ncRNAs are divided into various types including microRNAs (miRNAs), PIWI-interacting RNAs (piRNAs), small nuclear RNAs (snRNAs), small nucleolar RNAs (snoRNAs), long ncRNAs (lncRNAs), circular RNAs (circRNAs), transfer RNAs (tRNAs), and ribosomal RNAs (rRNAs)^22,23^. NcRNAs participate in regulation of tumorigenesis via various biological processes such as chromatin modification, alternative splicing, competition with endogenous RNAs and interaction with proteins^24,25^. For example, *miR-675-5p* promoted the metastasis of colorectal cancer cells via modulation of P53^26^. Moreover, lncRNA *HOTAIR* served as an enhancer in epithelial-to-mesenchymal transition of breast cancer cells via competing with BRCA1^27^. In addition, *circFOXO3* enhanced progression of prostate cancer through sponging *miR-29a-3p*^28^. However, roles of ncRNAs in ferroptosis have not been fully determined.

In this review, we focus on summarizing the ncRNAs which have been found to associate with ferroptosis regulators GSH, iron, nuclear factor (erythroid-derived 2)-like 2 (NRF2) and ROS in cancer^5^. Moreover, we predict the obstacles that may limit the exploration of ncRNAs in ferroptosis in cancer therapy and offer advice for future studies. We believe that a comprehensive understanding of the interactions between ncRNAs and ferroptosis may benefit clinical therapeutics to cancer

### MiRNAs and ferroptosis

MiRNAs exhibit functions by binding to the 3′-untranslated regions of target mRNAs and suppressing their expression^29^. Some studies have revealed a relationship between miRNAs and ferroptosis. In radioresistant cells, *miR-7-5p* inhibited ferroptosis via downregulating mitoferrin and thus reducing iron levels^30^. Furthermore, *miR-9* and *miR-137* enhanced ferroptosis via reduction of intracellular GSH levels, *miR-9* inhibited synthesis of GSH and *miR-137* suppressed solute carrier family 1 member 5 (SLC1A5), a component of systemXc^−^^31^. Moreover, *miR-6852* which was regulated by lncRNA *Linc00336*, inhibited growth of lung cancer cells via promoting ferroptosis^32^. In the following sections, we will discuss the interactions between miRNAs and GSH, iron and NRF2 in cancer cells. The information of altered miRNAs in ferroptosis has been listed (Supplementary Table 1).

### MiRNAs and GSH

**Fig. 1:**
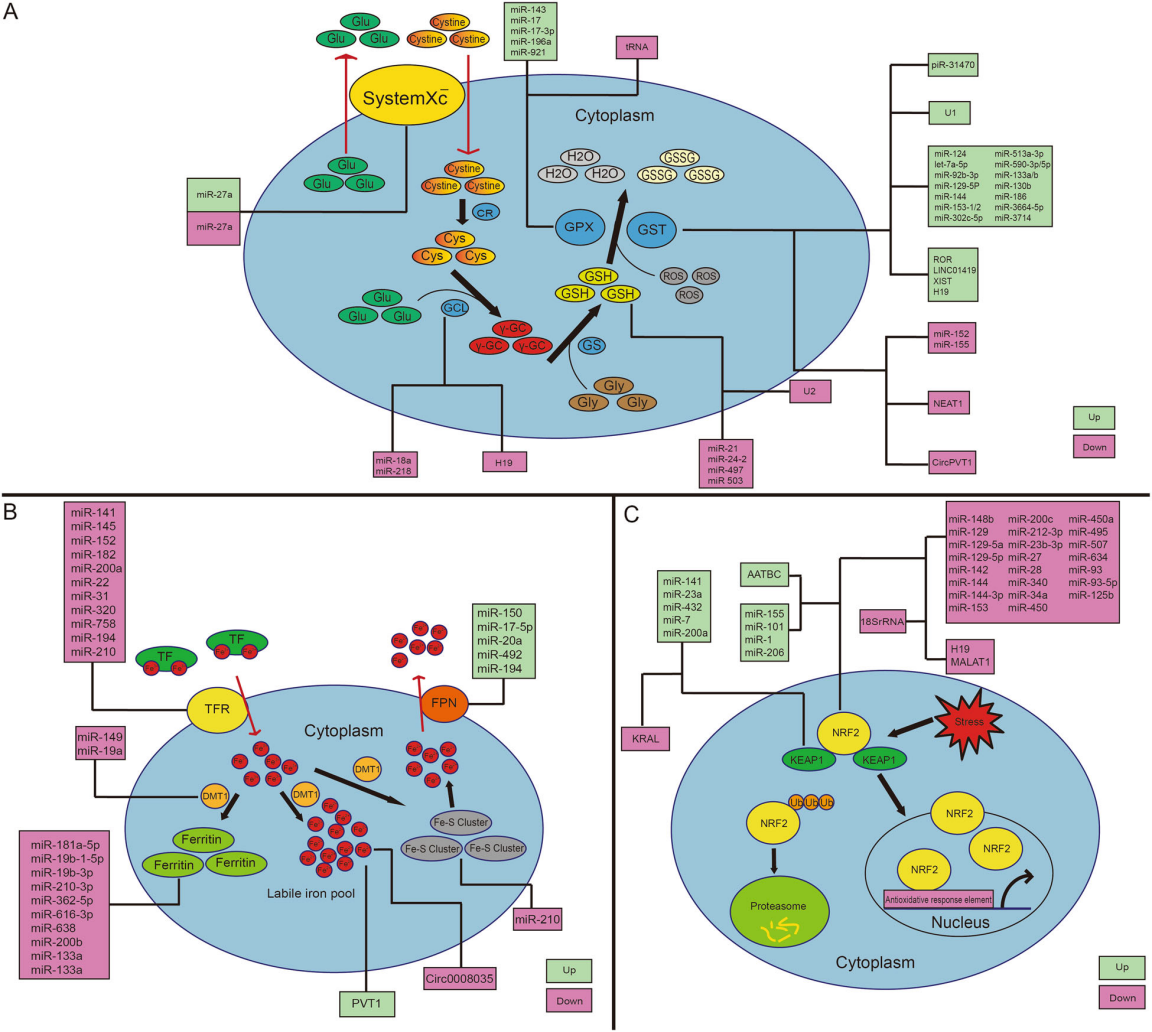
Regulation of ncRNAs to ferroptosis. **a** Regulation of ncRNAs to GSH metabolism; **b** Regulation of ncRNAs to iron metabolism; **c** Regulation of ncRNAs to KEAP1-NRF2 pathway.

GSH is a scavengerof ROS and protects lipid membrane^33^. Under physiological conditions, concentration of reduced GSH is about 10–100-fold more prevalent than the oxidized form. Under oxidative stress, reduced GSH is converted to oxidized form^34^. Biosynthesis of GSH involves three steps: exchange of glutamic acid and cystine induced by systemXc^−^; synthesis of 𝛾-glutamylcysteine by glutamic acid and cysteine catalyzed via 𝛾-glutamylcysteine ligase (GCL); and synthesis of GSH via 𝛾-glutamylcysteine and glycine catalyzed by GSH synthetase^35^. Function of GSH includes detoxification of exogenous or endogenous dangerous compounds catalyzed by GSH-S-transferases (GSTs) and GPXs^36^. Current knowledge on relation between GSH and cancer are summarized in Table 1, and the schematic diagram of these interactions is shown in Fig. 1a. *MiR-18a* and *miR-218* decreased GSH levels via targeting GCL in hepatocellular carcinoma and bladder cancer^37,38^. Furthermore, in hepatocellular carcinoma and lung cancer, *miR-152* and *miR-155* decreased GSH levels via targeting GST^39,40^. In addition, *miR-326* and *miR-27a* inhibited GSH levels in cancer cells via targeting other factors such as pyruvate kinase m 2 (PKM2), SLC7A11 and zinc finger and BTB domain containing 10 (ZBTB10)^41–43^. Additionally, downregulation of GSH by miRNAs such as *miR-21*, *miR-24-2*, *miR-497* and *miR‑503* has been observed in different cancer types, however, the specific mechanisms were not explored^44–47^. These findings indicate that miRNAs repress GSH levels via control of synthesis and consumption. The upregulation of GSH induced by miRNAs has been well-explored. GST was targeted by different miRNAs including *miR-124*, *let-7a-5p*, *miR-92b-3p*, *miR-129-5P*, *miR-144*, *miR-153-1/2*, *miR-302c-5p*, *miR-3664-5p*, *miR-3714*, *miR-513a-3p*, *miR-590-3p/5p*, *miR-130b*, *miR-186*, and *miR-133a/b*. These miRNAs bound to the 3′-untranslated regions of GST mRNA and inhibited GST expression, thus blocking GSH consumption and resulting in accumulation of intracellular GSH^48–51^. It is worth mentioning that *miR-133a/b* served as effective suppressors of GST in different cancer types, such as bladder cancer, lung cancer, prostate cancer, colorectal cancer, ovarian cancer and head and neck carcinoma. Inhibition of *miR-133a/b* reversed both increased GSH and insensitivity to drugs^51–54^. Furthermore, GPX family members are targeted by miRNAs and results in defect of ROS neutralization. In one report, GPX4 was decreased by *miR-181a-5p* in osteoarthritis^55^. However, the relationship between GPX4 and miRNAs in cancer is still in dark. Only GPX2 and GPX3 have been found to be modulated by miRNAs such as *miR-17*, *miR-17-3p*, *miR-196a*, and *miR-921* in colorectal cancer, prostate cancer, and lung cancer^56–59^. Overall, regulation of GSH by miRNAs occurs mainly through control of GST and GPX family members. Since GSH has been shown to participate in growth of tumors and chemoresistance to drugs which induce intracellular oxidative stress, miRNAs may regulate ferroptosis and control cancer progression via modulation of GSH.

**Table 1:** Summary of GSH associated miRNAs in cancer.

Name	Associated cancer type	Target	Influence to GSH	Model of evidence	Reference
*miR-27a*	Bladder cancer, colorectal cancer	SLC7A11, ZBTB10	Up/Down	Cell culture, animal models	^42,43^
*miR-143*	Colorectal cancer	GPX	Up	Animal models	^199^
*miR-17*	Prostate cancer	GPX2	Up	Cell culture, animal models	^56^
*miR-17-3p*	Prostate cancer	GPX2	Up	Cell culture, animal models	^57^
*miR-196a*	Lung cancer	GPX3	Up	Cell culture, animal models	^58^
*miR-921*	Lung cancer	GPX3	Up	Cell culture	^59^
*miR-124*	Colorectal cancer	GST	Up	Cell culture, animal models	^48^
*Let-7a-5p*	Prostate cancer	GST	Up	Cell culture, animal models	^49^
*miR-92b-3p*	Prostate cancer	GST	Up	Cell culture, animal models	^49^
*miR-129-5P*	Colorectal cancer cells	GST	Up	Cell culture	^50^
*miR-144*	Prostate cancer	GST	Up	Cell culture, animal models	^51^
*miR-153-1/2*	Prostate cancer	GST	Up	Cell culture, animal models	^51^
*miR-302c-5p*	Colorectal cancer	GST	Up	Cell culture	^50^
*miR-3664-5p*	Colorectal cancer	GST	Up	Cell culture	^50^
*miR-3714*	Colorectal cancer	GST	Up	Cell culture	^50^
*miR-513a-3p*	Colorectal cancer, lung cancer	GST	Up	Cell culture	^200^
*miR-590-3p/5p*	Prostate cancer	GST	Up	Cell culture, animal models	^51^
*miR-133a/b*	Bladder cancer, lung cancer, prostate cancer, colorectal cancer, ovarian cancer, head and neck squamous cell carcinoma	GST	Up	Cell culture, animal models	^52,53,201^
*miR-130b*	Ovarian cancer	GST	Up	Cell culture	^202^
*miR-186*	Ovarian cancer	GST	Up	Cell culture	^203^
*miR-34b*	Prostate cancer	MYC	Up	Cell culture	^204^
*miR-K12-11*	Kaposi’s sarcoma	xCT	Up	Cell culture	^205^
*miR-18a*	Hepatocellular carcinoma	GCL	Down	Cell culture, animal models	^37^
*miR-218*	Bladder cancer	GCL	Down	Cell culture	^38^
*miR-21*	Lung cancer	GSH	Down	Cell culture	^44^
*miR-24-2*	Colorectal cancer	GSH	Down	Clinical samples	^45^
*miR-497*	Cervical cancer	GSH	Down	Cell culture	^46^
*miR-503*	Hepatocellular carcinoma	GSH	Down	Cell culture	^47^
*miR-152*	Hepatocellular carcinoma	GST	Down	Cell culture	^39^
*miR-155*	Lung cancer	GST	Down	Cell culture	^40^
*miR-326*	Glioma	PKM2	Down	Cell culture	^41^
*miR-125b*	Chronic lymphocytic leukemias	GSH	Unknown	Cell culture	^206^

### MiRNAs and iron

Iron metabolism is another key factor in ferroptosis. Excessive iron increases ROS via Fenton reaction and ROS is neutralized by iron reversely^60^. Metabolism of iron mainly includes interaction between transferrin (TF) and its receptor (TFR), import of iron via divalent metal transporter 1 (DMT1), storage of iron as ferritin and iron-sulfur cluster (ISC), and export of iron via ferroportin (FPN)^61,62^. The specific realtion between miRNAs and iron is summarized in Table 2, and the schematic diagram of these interactions are shown in Fig. 1b. In colorectal cancer, targeting of DMT1 by *miR-149* and *miR-19a* led to decreased iron import^63^. Furthermore, in colorectal cancer and hepatocellular cancer, TFR was targeted by miRNAs including *miR-141*, *miR-145*, *miR-152*, *miR-182*, *miR-200a*, *miR-22*, *miR-31*, *miR-320*, *miR-758*, and *miR-194*^63–65^. This inhibition led to disruption of interaction between TF and TFR and the following decreased iron import. Thereinto, *miR-194* suppressed the expression of both TFR and FPN in colorectal cancer^63^. FPN was also targeted by *miR-150*, *miR-17-5p*, *miR-20a*, and *miR-492* in hepatocellular carcinoma, multiple myeloma, lung cancer, and prostate cancer, respectively^66–68^. Furthermore, ferritin which is composed of ferritin heavy chain (FHC) and ferritin light chain (FLC), is controlled by miRNAs^69^. FHC could be targeted by *miR-200b*, *miR-181a-5p*, *miR-19b-1-5p*, *miR-19b-3p*, *miR-210-3p*, *miR-362-5p*, *miR-616-3p*, and *miR-638* in prostate cancer, resulting in decreased intracellular iron^65,70,71^. FLC could be targeted by *miR-133a* in colorectal cancer and breast cancer, and knockdown of *miR-133a* restored the reduced iron levels inside cancer cells^63,72^. Among the miRNAs that regulate iron levels, *miR-210* serves as an important member. In colorectal cancer cells, *miR-210* was activated by hypoxia and then targeted ISCU to alter intracellular iron homeostasis^73^. Furthermore, transfection of *miR-210* decreased the uptake of iron via TFR suppression^74^. On the contrary, miRNAs can be modulated by iron. *MiR-107*, *miR-125b*, and *miR-30d* were inhibited by iron in hepatocellular carcinoma and ovarian cancer^75,76^, and *miR-146a*, *miR-150*, *miR-214-3p* and *miR-584* were increased by iron in ovarian cancer and neuroblastoma^76,77^. This phenomenon may derive from the induction of excess ROS by iron and the subsequent regulation of miRNAs transcription. Overall, different miRNAs regulate iron levels in various directions, and the imbalance of iron leads to run-away miRNA expression.

**Table 2:** Summary of iron associated miRNAs in cancer.

Name	Associated cancer type	Target	Influence to iron	Model of evidence	Reference
*miR-150*	Hepatocellular carcinoma	FPN	Up	Cell culture	^76^
*miR-17-5p*	Multiple myeloma	FPN	Up	Cell culture, animal models	^66^
*miR-20a*	Lung cancer	FPN	Up	Cell culture	^67^
*miR-492*	Prostate cancer	FPN	Up	Cell culture, animal models	^68^
*miR-194*	Colorectal cancer	TFR1, FPN1	Up	Clinical samples	^63^
*miR-449a*	Glioma	CDGSH iron sulfur domain 2	Down	Cell culture, animal models	^207^
*miR-149*	Colorectal cancer	DMT1	Down	Clinical samples	^63^
*miR-19a*	Colorectal cancer	DMT1	Down	Clinical samples	^63^
*miR-181a-5p*	Prostate cancer	FHC	Down	Cell culture, animal models	^70^
*miR-19b-1-5p*	Prostate cancer	FHC	Down	Cell culture, animal models	^70^
*miR-19b-3p*	Prostate cancer	FHC	Down	Cell culture, animal models	^70^
*miR-210-3p*	Prostate cancer	FHC	Down	Cell culture, animal models	^70^
*miR-362-5p*	Prostate cancer	FHC	Down	Cell culture, animal models	^70^
*miR-616-3p*	Prostate cancer	FHC	Down	Cell culture, animal models	^70^
*miR-638*	Prostate cancer	FHC	Down	Cell culture, animal models	^70^
*miR-200b*	Hepatocellular carcinoma, breast cancer	Ferritin	Down	Cell culture	^65,71^
*miR-133a*	Colorectal cancer, breast cancer	FLC	Down	Cell culture	^63,72^
*miR-29*	Lung cancer	Iron-responsive element binding protein 2	Down	Clinical samples	^208^
*miR-210*	Renal cancer, head and neck paragangliomas, breast cancer, colorectal cancer, and oropharyngeal squamous cell carcinomas	ISCU, TFR1	Down	Cell culture, animal models	^73,74,209–211^
*miR-126*	Malignant mesothelioma	Mitochondria-destabilizing stress signals	Down	Cell culture, animal models	^212^
*miR-7-5p*	Ovarian cancer, colorectal cancer	Mitoferrin	Down	Cell culture	^30^
*miR-122*	Hepatocellular cancer	Nocturnin	Down	Cell culture, animal models	^213^
*miR-34a*	Lung cancer	P53	Down	Cell culture, animal models	^214^
*miR-141*	Colorectal cancer	TFR1	Down	Clinical samples	^63^
*miR-145*	Colorectal cancer	TFR1	Down	Clinical samples	^63^
*miR-152*	Hepatocellular carcinoma	TFR1	Down	Cell culture, animal models	^64^
*miR-182*	Colorectal cancer	TFR1	Down	Clinical samples	^63^
*miR-200a*	Hepatocellular carcinoma	TFR1	Down	Cell culture	^65^
*miR-22*	Hepatocellular cancer	TFR1	Down	Cell culture	^65^
*miR-31*	Colorectal cancer	TFR1	Down	Clinical samples	^63^
*miR-320*	Hepatocellular cancer	TFR1	Down	Cell culture	^65^
*miR-758*	Colorectal cancer	TFR1	Down	Clinical samples	^63^
*miR-107*	Hepatocellular carcinoma	–	Inhibited by iron	Cell culture, animal models	^75^
*miR-125b*	Ovarian cancer	–	Inhibited by iron	Cell culture	^76^
*miR-30d*	Hepatocellular carcinoma	–	Inhibited by iron	Cell culture, animal models	^75^
*miR-146a*	Ovarian cancer	–	Induced by iron	Cell culture	^76^
*miR-150*	Ovarian cancer	–	Induced by iron	Cell culture	^76^
*miR-214-3p*	Neuroblastoma	–	Induced by iron	Cell culture	^77^
*miR-584*	Neuroblastoma	–	Induced by iron	Cell culture	^77^

### MiRNAs and NRF2

NRF2 serves as a transcriptional factor and activates downstream antioxidant factors. The expression of NRF2 mainly depends on Kelch-like ECh-Associated Protein 1 (KEAP1), which assembles Cullin3 to form the Cullin-E3 ligase complex and then degrades NRF2 protein via the ubiquitin-proteasome route^78^. Inhibition of NRF2 has been confirmed to enhance ferroptosis^79^. The specific information regarding interaction between miRNAs and NRF2 is listed in Table 3, and the schematic diagram is shown in Fig. 1c. In esophageal cancer, *miR-129*, *miR-142*, *miR-144-3p*, *miR-450*, *miR-507*, and *miR-634* targeted the 3′-untranslated region of NRF2 mRNA and decreased NRF2 expression, resulting in an increase of ROS^80–85^. Among these miRNAs, *miR-144-3p* played an important role in the regulation of NRF2. Targeting NRF2 by *miR-144-3p* inhibited tumor progression in melanoma and acute myeloid leukemia^86^, and increased the sensitivity of lung cancer cells to cisplatin^87^, indicating the role of *miR‑144‑3p* in oxidative homeostasis. Other miRNAs that targeted NRF2 include *miR-144*, *miR-153*, *miR-200c*, and *miR-212-3p*, although their effects have not been explored^82,88–90^. Moreover, miRNAs regulate NRF2 via targeting KEAP1. In hepatocellular carcinoma, ovarian cancer, leukemia, and neuroblastoma cells, KEAP1 was targeted by *miR-141*, *miR-23a*, *miR-432*, *miR-7*, and *miR-200a*^88,91–95^. Thereinto, *miR-200a* served as an active role. In esophageal squamous cell carcinoma, methylseleninic acid activated KEAP1/NRF2 pathway via upregulating *miR-200a*, the latter inhibited KEAP1 expression and induced expression of NRF2^96^. In breast cancer and pancreatic adenocarcinoma, *miR-200a* suppression reverted expression of KEAP1 and then inhibited NRF2 and promoted the anchorage-independent cell growth in vitro^97^. In turn, NRF2 enhances miRNAs expression via binding to the antioxidative response element box. In myelocytic leukemia, *miR-125b* driven by NRF2 promoted leukemic cells survival. Inhibition of *miR-125b* enhanced responsiveness of leukemic cells towards chemotherapy^98^. However, in oral squamous cell carcinoma, repression of *miR-125b* by peroxiredoxin like 2A (PRXL2A) protected cancer cells from drug-induced oxidative stress in an NRF2-depedent manner^99^, indicating the mutual regulation between *miR-125b* and NRF2. In addition, expression of *miR-29B1*, *miR-129-3p*, and *miR-380-3p* was induced by NRF2 in acute myelocytic leukemia, hepatocellular carcinoma, and neuroblastoma^98,100,101^. Conversely, *miR-181c*, *miR-378*, *miR-122*, *miR-17-5p*, *miR-1*, and *miR-206* were repressed by NRF2 in various cancer types^66,102–107^. Thereinto, inhibition of *miR-1* and *miR-206* was mediated by SOD1 induced by NRF2 but not the role of NRF2 as a transcriptional factor. In summary, miRNAs regulate NRF2 pathway through targeting KEAP1 and NRF2 mRNAs. Conversely, NRF2 controls miRNAs via transcription or downstream factor SOD1.

**Table 3:** Summary of NRF2 associated miRNAs in cancer.

Name	Associated cancer type	Target	Influence to NRF2	Model of evidence	Reference
*miR-141*	Hepatocellular carcinoma, ovarian cancer	KEAP1	Up	Cell culture	^88,91–93^
*miR-23a*	Leukemic	KEAP1	Up	Cell culture, animal models	^94^
*miR-432*	Esophageal cancer	KEAP1	Up	Cell culture	^92,95^
*miR-7*	Neuroblastoma cells	KEAP1	Up	Cell culture	^92^
*miR-200a*	Breast cancer, esophageal cancer, hepatocellular carcinoma, and pancreatic adenocarcinomas	KEAP1,	Up	Cell culture, animal models	^81,92,96,97,215,216^
*miR-155*	Lung cancer	NRF2	Up	Cell culture	^217^
*miR-101*	Hepatocellular carcinoma, prostate cancer	NRF2, SOD1	Up/Down	Cell culture, animal models	^88,105,218^
*miR-1*	Lung cancer, prostate cancer	NRF2, SOD1	Up/Inhibited by NRF2	Cell culture, animal models	^105,107^
*miR-206*	Lung cancer, prostate cancer	NRF2, SOD1	Up/Inhibited by NRF2	Cell culture, animal models	^105–107^
*miR-148b*	Endometrial cancer	ERMP1	Down	Cell culture	^219^
*miR-129*	Esophageal cancer	NRF2	Down	Cell culture, animal models	^80^
*miR-129-5a*	Esophageal cancer	NRF2	Down	Cell culture, animal models	^80,81^
*miR-129-5p*	Esophageal cancer	NRF2	Down	Cell culture, animal models	^80^
*miR-142*	Esophageal cancer	NRF2	Down	Cell culture	^82^
*miR-144*	Hepatocellular carcinoma, leukemia, hepatocellular carcinoma, neuroblastoma	NRF2	Down	Cell culture	^88,89^
*miR-144-3p*	Melanoma, lung cancer, and acute myeloid leukemia	NRF2	Down	Cell culture	^86,87,220,221^
*miR-153*	Neuroblastoma, breast cancer, and oral squamous cell carcinoma	NRF2	Down	Cell culture	^82,90^
*miR-200c*	Lung cancer	NRF2	Down	Cell culture, animal models	^222^
*miR-212-3p*	Melanoma	NRF2	Down	Cell culture	^86^
*miR-23b-3p*	Melanoma	NRF2	Down	Cell culture	^86^
*miR-27*	Neuroblastoma	NRF2	Down	Cell culture, animal models	^223^
*miR-28*	Breast cancer, esophageal cancer	NRF2	Down	Cell culture	^81,224^
*miR-340*	Hepatocellular carcinoma, esophageal cancer	NRF2	Down	Cell culture	^85,88,92^
*miR-34a*	Breast cancer, colon cancer, ovarian cancer, and lung cancer	NRF2	Down	Cell culture	^225,226^
*miR-450*	Esophageal cancer	NRF2	Down	Cell culture	^83^
*miR-450a*	Esophageal cancer	NRF2	Down	Cell culture, animal models	^80,81^
*miR-495*	Nonsmall-cell lung cancer	NRF2	Down	Cell culture	^227^
*miR-507*	Esophageal cancer	NRF2	Down	Cell culture, animal models	^80,81,84^
*miR-634*	Esophageal cancer	NRF2	Down	Cell culture, animal models	^80,81,85^
*miR-93*	Pancreatic adenocarcinomas, breast cancer	NRF2	Down	Cell culture, animal models	^97,221^
*miR-93-5p*	Melanoma	NRF2	Down	Clinical samples	^86^
*miR-125b*	Acute myelocytic leukemia, oral squamous cell carcinoma, and renal cancer	NRF2	Down/Induced by NRF2	Cell culture, animal models	^98,99,228^
*miR-181c*	Colorectal cancer	–	Inhibited by NRF2	Cell culture, animal models	^102^
*miR-378*	Mucoepidermoid carcinoma	–	Inhibited by NRF2	Cell culture, animal models	^103^
*miR-122*	Hepatocellular carcinoma	–	Inhibited by NRF2	Cell culture	^104^
*miR-17-5p*	Multiple myeloma	–	Inhibited by NRF2	Cell culture, animal models	^66^
*miR-29B1*	Acute myelocytic leukemia	–	Induced by NRF2	Cell culture	^98^
*miR-129-3p*	Hepatocellular carcinoma	–	Induced by NRF2	Cell culture, animal models	^100^
*miR-380-3p*	Neuroblastoma	–	Induced by NRF2	Cell culture, animal models	^101^

### MiRNAs and ROS

In addition to factors above, miRNAs regulate ROS via other mechanisms. The information of miRNAs that are related to ROS in cancer is listed in Table 4. MiRNAs can positively regulate ROS levels. For example, *miR-21* whose expression increased with tumor grade, has been identified to enhance ROS level in lung cancer, colorectal cancer, gastric cancer, hepatocellular carcinoma, ovarian cancer, and prostate cancer^108–113^. Mechanically, *miR-21* targeted STAT3, proline oxidase (POX), and programmed cell death 4 (PDCD4) to induce oxidative stress^114–116^. Moreover, *miR-146a* has attracted much attention. In ovarian cancer, *miR-146a* repressed SOD2 expression and inhibited proliferation of cancer cells and enhanced chemosensitivity to drugs^117^. In lung cancer, suppression of *miR-146a* restored catalase and inhibited ROS induction, and protected cancer cells from cisplatin-induced cytotoxicity^118^. In addition, overexpression of *miR-124*, *miR-526b*, and *miR-655* led to excess ROS via thioredoxin reductase 1 in breast cancer^119,120^. Furthermore, the antioxidant enzyme SOD1 was downregulated by stable expression of *miR-143* or *miR-145* in colorectal cancer^121^. This indicates that miRNAs enhance intracellular ROS via different manners. On the other hand, in lung cancer, *miR-99* suppressed the invasion and migration of cancer cells via targeting NOX4-mediated ROS production^122^. Additionally, *miR-520* and *miR-373* reduced ROS via targeting NF-κB and TGF-β signaling pathways and repressed growth and lymph node metastasis of breast cancer^123^. Other miRNAs such as *let-7*, *miR-137*, *miR-193b*, *miR‑199*, and *miR-26a*, have been found to decrease ROS level in cancer cells via diverse targets such as heme oxygenase-1, C-MYC, and triglyceride^124–128^, indicating that miRNAs inhibit ROS level. Conversely, *miR-133a*, *miR-150-3p*, *miR-1915-3p*, *miR-206*, *miR-34*, *miR-638*, and *miR-182* were activated by oxidative stress and then played a role in the subsequent biological processes^129–133^. Moreover, *miR-125*, *miR-145-5p*, *miR-17-5p*, *miR-199*, and *miR-17-92*, were decreased by excess intracellular ROS^134–137^. Among them, *miR-125b* plays a dual role in oxidative homeostasis. As discussed above, *miR-125b* serves as a regulator of NRF2. In addition, *miR-125b* could be inhibited by ROS via a DNMT1-dependent DNA methylation in ovarian cancer^140^. Moreover, although *miR-21* has been discussed as the enhancer of ROS in breast cancer, DNA damage induced by ROS led to activation of *miR-21* via NF-κB, indicating the interaction between miRNAs and ROS^138^. In total, we can infer that altered levels of GSH, iron, and NRF2 are not the only methods by which miRNAs regulate ROS and vice versa in, miRNAs and ROS can also regulate each other in various pathways.

**Table 4:** Summary of ROS associated miRNAs in cancer.

Name	Associated cancer type	Target	Influence to ROS	Model of evidence	Reference
*miR-124*	Non-small cell lung cancer	TXNRD1	Up	Cell culture	^120^
*miR-125a*	Osteosarcoma	Estrogen-related receptor alpha	Up	Cell culture	^229^
*miR-128a*	Medulloblastoma	BMI-1	Up	Cell culture	^230^
*miR-139-5p*	Breast cancer	Unknown	Up	Cell culture, animal models	^231^
*miR-143*	Colorectal cancer	SOD1	Up	Cell culture	^121^
*miR-146a*	Lung cancer, ovarian Cancer	Catalase, SOD2	Up	Cell culture, animal models	^117,118^
*miR-146b-5p*	Leukemic	Unknown	Up	Cell culture	^232^
*miR-15*	Colorectal cancer, cancer stem cells	C-MYC	Up	Cell culture, animal models	^233^
*miR-155*	Glioma, pancreatic cancer	MAPK13, MAPK14, and Foxo3a	Up	Cell culture, animal models	^234,235^
*miR-15a-3p*	Lung cancer	P53	Up	Cell culture	^236^
*miR-16*	Colorectal cancer, cancer stem cells	C-MYC	Up	Cell culture, animal models	^233^
*miR-186*	Colorectal cancer	CKII	Up	Cell culture	^237^
*miR-193a-3p*	Glioma	γH2AX	Up	Cell culture	^238^
*miR-210*	Cancer stem cells, glioma	P53	Up	Cell culture, animal models	^239^
*miR-212*	Colorectal cancer	MnSOD	Up	Clinical samples	^240^
*miR-216b*	Colorectal cancer	CKII	Up	Cell culture	^237^
*miR-22*	Hepatocellular carcinoma	SIRT-1	Up	Cell culture	^241^
*miR-223*	Breast cancer	HAX-1	Up	Cell culture	^242^
*miR-23b-3p*	Acute myeloid leukemia	PrxIII	Up	Cell culture	^243^
*miR-25-5p*	Colorectal cancer	SOX10	Up	Cell culture	^244^
*miR-26a-5p*	Acute myeloid leukemia	PrxIII	Up	Cell culture	^243^
*miR-26b*	Small cell lung cancer	Myeloid cell leukemia 1 protein	Up	Cell culture, animal models	^245^
*miR-30*	Gastric cancer	P53	Up	Cell culture	^246^
*miR-337-3p*	Colorectal cancer	CKII	Up	Cell culture	^237^
*miR-34c*	Nonsmall cell lung cancer	HMGB1	Up	Cell culture	^247^
*miR-371-3p*	Lung cancer	PRDX6	Up	Cell culture, animal models	^248^
*miR-422a*	Gastric cancer	PDK2	Up	Cell culture, animal models	^249^
*miR-4485*	Breast cancer	Mitochondrial protein	Up	Cell culture, animal models	^133^
*miR-4673*	Lung cancer	8-Oxoguanine-DNA Glycosylase-1	Up	Cell culture	^250^
*miR-504*	Lung cancer	P53	Up	Cell culture	^251^
*miR-506*	Lung cancer	P53, NF-κB	Up	Cell culture, animal models	^252^
*miR-509*	Breast cancer	P53	Up	Cell culture	^253^
*miR-526b*	Breast cancer	Thioredoxin Reductase 1	Up	Cell culture	^119^
*miR-551b*	Lung cancer	MUC1	Up	Cell culture	^254^
*miR-655*	Breast cancer	Thioredoxin Reductase 1	Up	Cell culture	^119^
*miR-661*	Colorectal cancer	Hexose-6-phosphate dehydrogenase, pyruvate kinase M2	Up	Cell culture	^255^
*miR-760*	Colorectal cancer	CKII	Up	Cell culture	^237^
*miR-92*	Hepatocellular carcinoma	Unknown	Up	Clinical samples	^256^
*miR-128*	Glioma, hepatocellular carcinoma	PKM2	Up/Down	Cell culture	^257^
*miR-145*	Colorectal cancer, hepatocellular carcinoma	SOD1, PKM2	Up/Down	Cell culture	^121,258^
*miR-211*	Myeloma, oral carcinoma	PRKAA1, TCF12	Up/Down	Cell culture, animal models	^259,260^
*miR-222*	Hepatocellular carcinoma, breast cancer	NF-κB, TGF-β	Up/Down	Cell culture, animal models	^261,262^
*miR-23a/b*	Myeloma, renal cancer	C-MYC, POX	Up/Down	Cell culture, animal models	^263,264^
*miR-29*	Ovarian cancer, lung cancer, and lymphoma	C-MYC, SIRT1	Up/Down	Cell culture, animal models	^265,266^
*miR-34a*	Gastric cancer, glioma	NOX2	Up/Down	Cell culture	^267^
*Let-7*	Hepatocellular carcinoma, prostate cancer, and pancreatic cancer	Heme oxygenase-1, P53	Up/Down	Cell culture, animal models	^123,268^
*miR-33a*	Glioma, hepatocellular carcinoma	SIRT6	Up/Down	Cell culture, animal models	^269^
*miR-221*	Hepatocellular carcinoma, breast cancer	NF-κB, TGF-β, and DICER	Up/Down/Induced by ROS	Cell culture, animal models	^261,262,270^
*miR-21*	Lung cancer, colorectal cancer, gastric cancer, hepatocellular carcinoma, ovarian cancer, and prostate cancer	SOD, MAPK, SOD2, Glucose, NFκB, STAT3, POX, and PDCD4	Up/Down/Induced by ROS	Cell culture, animal models	^108,112,114,271^
*miR-17-92*	Gastric cancer, lung cancer	C-MYC, P53, and NFκB	Up/Down/Inhibited by ROS	Cell culture	^137,272,273^
*miR-181*	Hepatocellular carcinoma, uterine leiomyoma	Unknown	Up/Induced by ROS	Cell culture	^132,274^
*miR-200*	Breast cancer, cancer stem cells, hepatocellular carcinoma, and lung cancer	P53, PRDX2, GAPB/NRF2, SESN1	Up/Induced by ROS	Cell culture, animal models	^222,275–277^
*miR-34*	Cancer stem cells, bladder cancer, lung cancer	C-MYC, P53	Up/Induced by ROS	Cell culture	^278,279^
*miR-182*	Uterine leiomyoma, lung cancer	PDK4	Up/Induced by ROS	Cell culture, animal models	^132,133^
*miR-199*	Gastric cancer, ovarian cancer	DNMT1	Up/Inhibited by ROS	Cell culture	^134^
*miR-20a*	Breast cancer, pancreatic cancer	BECN1, ATG16L1, and SQSTM1	Up/Inhibited by ROS	Cell culture, animal models	^136,280^
*miR-125b*	Hepatocellular carcinoma, ovarian cancer, and breast cancer	Hexokinase 2, DNMT1, and HAX-1	Up/Inhibited by ROS	Cell culture, animal models	^228,281^
*miR-1246*	Breast cancer	NF-κB, TGF-β	Down	Cell culture	^124^
*miR-137*	Ovarian cancer	C-MYC	Down	Cell culture, animal models	^125^
*miR-193b*	Liposarcoma	Antioxidant methionine sulfoxide reductase A	Down	Cell culture, animal models	^127^
*miR-199a-3p*	Testicular cancer	Transcription factor specificity protein 1	Down	Cell culture	^126^
*miR-26a*	Hepatocellular carcinoma	Triglyceride, totalcholesterol, malondialdehyde	Down	Cell culture	^128^
*miR-30c-2-3p*	Breast cancer	NF-κB, TGF-β	Down	Cell culture	^282^
*miR-346*	Ovarian cancer	GSK3B	Down	Cell culture	^283^
*miR-373*	Breast cancer	NF-κB, TGF-β	Down	Cell culture, animal models	^123^
*miR-520*	Breast cancer	NF-κB, TGF-β	Down	Cell culture, animal models	^123^
*miR-7*	Nonsmall cell lung cancer	MAFG	Down	Cell culture	^284^
*miR-885-5p*	Hepatocellular carcinoma	TIGAR	Down	Cell culture	^285^
*miR-99a*	Lung cancer	NOX4	Down	Cell culture, animal models	^122^
*miR-133a*	Rhabdomyosarcoma	9	Induced by ROS	Cell culture, animal models	^129^
*miR-150-3p*	Hepatocellular carcinoma	–	Induced by ROS	Cell culture	^130^
*miR-1915-3p*	Hepatocellular Carcinoma	–	Induced by ROS	Cell culture	^130^
*miR-206*	Rhabdomyosarcoma	–	Induced by ROS	Cell culture, animal models	^131^
*miR-34a-3p*	Hepatocellular carcinoma	–	Induced by ROS	Cell culture, animal models	^129^
*miR-34a-5p*	Hepatocellular carcinoma	–	Induced by ROS	Cell culture	^130^
*miR-638*	Hepatocellular carcinoma	–	Induced by ROS	Cell culture	^130^
*miR-125*	Gastric cancer	–	Inhibited by ROS	Cell culture	^134^
*miR-145-5p*	Gastric cancer	–	Inhibited by ROS	Cell culture, animal models	^135^
*miR-17-5p*	Pancreatic cancer	–	Inhibited by ROS	Cell culture, animal models	^136^
*miR-27a*	Pancreatic cancer, colorectal cancer	–	Inhibited by ROS	Cell culture, animal models	^286^
*miR-328*	Gastric cancer	–	Inhibited by ROS	Cell culture, animal models	^287^
*miR-329*	Breast cancer	–	Inhibited by ROS	Cell culture, animal models	^288^
*miR-362-3p*	Breast cancer	–	Inhibited by ROS	Cell culture, animal models	^288^

### LncRNAs and ferroptosis

LncRNAs mainly serve as regulators of transcription factors in nucleus or as sponges of miRNAs in cytoplasm^139^. *Linc00336* was promoted by lymphoid-specific helicase in lung cancer and inhibited ferroptosis via sponging *miR-6852*^32^. Furthermore, in breast cancer and lung cancer, lncRNA *P53rra* bound to Ras GTPase-activating protein-(SH3domain)-Binding Protein 1 (G3BP1) and displaced P53 from a G3BP1 complex, resulting in retention of P53 in nucleus and downregulation of SLC7A11^140^. In addition, ferroptosis inducer erastin upregulated lncRNA GA binding protein transcription factor subunit beta 1 (GABPB1) antisense RNA 1 (*Gabpb1-AS1*), which suppressed GABPB1 and led to downregulation of peroxiredoxin-5 peroxidase and suppression of cellular antioxidant capacity in hepatocellular carcinoma^141^. Interaction between lncRNAs and ferroptosis has been listed (Supplementary Table 1), and the relationship between lncRNAs and ferroptosis associated factors is summarized in Table 5. The schematic diagram of these interactions is shown in Fig. 1.

**Table 5:** Summary of GSH, iron, NRF2, and ROS associated lncRNAs in cancer.

Control point	Name	Associated cancer type	Target	Influence to control point	Model of evidence	Reference
GSH	*Linc01419*	Esophageal squamous cell carcinoma	GST	Up	Clinical samples	^144^
	*Neat1*	Hepatocellular carcinoma	GST	Up	Cell culture	^143^
	*H19*	Ovarian cancer	GCLC, GCLM, GST	Up/Down	Cell culture, animal models	^145^
	*Xist*	Colorectal cancer	GST	Down	Cell culture, animal models	^48^
	*Ror*	Breast cancer	GST	Down	Cell culture, animal models	^142^
Iron	*Pvt1*	Hepatocellular carcinoma	*miR-150*/HIG2	Up	Cell culture, animal models	^146^
	*H19*	Myeloid leukemia	*miR-675*	Inhibited by iron	Cell culture	^147^
NRF2	*Aatbc*	Bladder cancer	NRF2	Down	Cell culture, animal models	^148^
	*Kral*	Hepatocellular carcinoma	KEAP1	Down	Cell culture	^91^
	*Malat1*	Multiple myeloma	KEAP1	Down	Cell culture, animal models	^149^
	*H19*	Ovarian cancer	NRF2	Down	Cell culture, animal models	^145^
	*Scal1*	Lung cancer	–	Induced by NRF2	Cell culture	^92^
	*Loc344887*	Gallbladder cancer	–	Induced by NRF2	Cell culture	^150^
ROS	*Meg3*	Lung cancer	P53	Up	Cell culture	^154^
	*Uca1*	Bladder cancer	*miR-16*	Down	Cell culture	^151^
	*Gas5*	Melanoma	G6PD	Down	Cell culture	^153^
	*H19*	Hepatocellular carcinoma	MAPK/ERK signaling pathway	Down	Cell culture	^152^
	*Miat*	Neuroblastoma, glioblastoma	MAPK7, FUT8, and MCL1	Unknown	Cell culture	^289–295^

### LncRNAs and ferroptosis associated factors

Since there are only a few studies about lncRNAs and ferroptosis factors, we will discuss them together. Regulation of GSH by lncRNAs in cancer mainly depends on GST and GCL^46^. In breast cancer, knockdown of lncRNA *Ror* led to reduced multidrug resistance-associated P-glycoprotein and GST expression, resulting in restored sensitivity of breast cancer cells to tamoxifen^142^. Similarly, in colorectal cancer, knockdown of lncRNA *Xist* inhibited doxorubicin resistance via suppressing GST and increasing GSH^48^. Furthermore, in hepatocellular carcinoma cells, silencing lncRNA *Neat1* inhibited IL-6-induced STAT3 phosphorylation which contributed to the increase of GST^143^. In addition, lncRNA *Linc01419* bound to the promoter region of *GSTP1* and recruited DNA methyltransferase, increasing promoter methylation and decreasing GST expression in esophageal squamous cell carcinoma^144^. Moreover, knockdown of lncRNA *H19* resulted in recovery of cisplatin sensitivity via reduction of GCL and GST^145^. In total, regulation of GSH by lncRNAs mainly depends on GST and GCL. Moreover, in hepatocellular carcinoma, silencing of lncRNA *Pvt1* inhibited TFR expression and obstructed iron uptake via *miR-150*^146^. Furthermore, silencing of FHC in leukemia cells induced production of ROS and altered downstream genes via increasing *H19* and *miR-657* expression^147^. This means that lncRNAs are associated with iron metabolism in cancer cells. Moreover, in bladder cancer, suppression of NRF2 by lncRNA associated transcript in bladder cancer (*Aatbc*) resulted in apoptosis^148^. In multiple myeloma, metastasis associated lung adenocarcinoma transcript 1 (*Malat1*) which has been proved to play a role in various cancers, inhibited NRF2 via activation of their negative regulator KEAP1^149^. Furthermore, overexpression of Keap1 regulation-associated lncRNA (*Kral*) inhibited NRF2 via increasing KEAP1 expression, and reversed the resistance of hepatocellular carcinoma cells to 5-fluorouracil^91^. Therefore, lncRNAs regulate NRF2 expression via direct and indirect manners. On the contrary, NRF2 participates in regulation of lncRNAs. In gallbladder cancer, downregulation of lncRNA *loc344887* suppressed cell proliferation and decreased migration and invasion. Further studies found that *loc344887* was upregulated after ectopic expression of NRF2^150^. In a recent study, NRF2 activated smoke and cancer-associated lncRNA 1 (*Scal1*) and induced oxidative stress protection. Knockdown of NRF2 suppressed *Scal1* and alleviated the proliferation of lung cancer cells^92^. In sum, lncRNAs can regulate NRF2 by directly controlling expression or modulating KEAP1 indirectly, and NRF2 can regulate lncRNAs expression reversely.

Other than the factors above, lncRNAs regulate ROS levels via various mechanisms. In bladder cancer, lncRNA urothelial cancer associated 1 (*Uca1*) decreased ROS level via targeting *miR-16* which led to decreased GSH synthetase^151^. Furthermore, in hepatocellular carcinoma, downregulation of *H19* increased ROS via MAPK/ERK signaling pathway and reversed chemotherapy resistance^152^. Moreover, knockdown of lncRNA growth arrest specific 5 (*Gas5*) in melanoma enhanced intracellular ROS via increased superoxide anion and NADPH oxidase 4 (NOX4)-oxidized GSH^153^. In lung cancer cells, the intracellular oxidative stress induced by paclitaxel was attenuated by knockdown of maternally expressed 3 (*Meg3*), and *Meg3* overexpression induced cell death and increased sensitivity to paclitaxel in an ROS-dependent manner^154^. In total, lncRNAs influence ROS metabolism via control of GSH, iron, NRF2 and other factors, and these factors can regulate lncRNAs expression reversely.

### Other ncRNAs and ferroptosis

CircRNAs, tRNAs, rRNAs, piRNAs, snRNAs, and snoRNAs are also contained in family of noncoding RNAs^21^. However, studies on the relations between these ncRNAs and ferroptosis are few. The interactions have been listed (Supplementary Table 2). The schematic diagram of these interactions is shown in Fig. 1.

### CircRNAs

CircRNAs are covalently closed, single-stranded RNA molecules derive from exons via alternative mRNA splicing^22^. Several studies have uncovered function of circRNAs in ferroptosis. In glioma, *circ-TTBK2* enhanced cell proliferation and invasion and inhibited ferroptosis via sponging *miR-761* and subsequent ITGB8 activation, knockdown of *circ-TTBK2* promoted erastin-induced ferroptosis^155^. Furthermore, *circ0008035* inhibited ferroptosis in gastric cancer via *miR-599*/EIF4A1 axis. Knockdown of *circ0008035* enhanced anticancer effect of erastin and RSL3 via increased iron accumulation and lipid peroxidation^156^. According to ferroptosis associated factors, in gastric cancer, *circPVT1* promoted multidrug resistance by enhancing P-gp and GSTP. MRNA levels of P-gp and GSTP were obviously repressed after downregulation of *circ-PVT1* in paclitaxel-resistant gastric cancer cells^157^. Moreover, high-throughout microarray-based circRNA profiling revealed that 526 circRNAs were dysregulated in cervical cancer cells, and bioinformatic analyses indicated that these circRNAs participated mainly in GSH metabolism^158^. However, associated miRNAs and downstream factors were not screened. Thus, further studies on the modulation of ferroptosis by circRNAs are needed.

### TRNAs

TRNAs serve as adapter molecules between mRNAs and proteins. The interaction between tRNAs and ferroptosis includes two possible manners. First, tRNAs are required in the synthesis of ferroptosis associated factors such as SLC7A11, GPX4, and IREB2, thus the mutation of tRNAs may alter the expression of these factors and then influence ferroptosis^217^. Second, tRNAs have multiple interaction partners including aminoacyl-tRNA-synthetases, mRNAs, ribosomes and translation factors^159^. Among them, cysteinyl-tRNA synthetase plays a role in ferroptosis. In fibrosarcoma, rhabdomyosarcoma and pancreatic carcinoma, loss of cysteinyl-tRNA synthetase suppressed erastin-induced ferroptosis via increasing intracellular GSH and transsulfuration, and inhibition of the transsulfuration pathway resensitized cells to erastin^160^. Interestingly, tRNAs mutation may control ferroptosis in an opposite manner. Selenocysteine which is formed from serine at the respective tRNA, is a component of GPXs. However, in hepatoma, colorectal cancer and breast cancer, the mutation of tRNA led to decline of selenoprotein expression except GPX4 and GPX1, and weak ferroptosis alteration^161–163^. This indicates that tRNAs modulate GSH levels mainly via synthesis but not metabolism. In addition, tRNAs influence ROS levels via various manners. Lung cancer mouse model with deletion of selenocysteine-tRNA gene exhibited ROS accumulation and increased susceptibility to lymph nodules metastasis^164^. Additionally, Queuine-modified tRNAs promoted cellular antioxidant defense via catalase, SOD, GPX, and GSH reductase and inhibited lymphoma^165^. In total, tRNAs decrease GSH synthesis and increase ferroptosis without modulating GPX4, while on the other hand, tRNAs enhance the antioxidant defense system and then inhibit ferroptosis.

### RRNAs

RRNAs constitute the structural and functional core of ribosomes^166^. Some reports have provided clues for role of rRNAs in ferroptosis. In cervical cancer, NRF2 was found to contain a highly conserved 18S rRNA binding site on 5′ untranslated region that is required for internal initiation. Deletion of this site remarkably enhanced translation, indicating that the 18S rRNA regulates NRF2 expression^167^. In another study, hepatoma cells treated with ethidium bromide exhibited a 70% decrease in the 16S/18S rRNA ratio and enhanced NRF2 expression^168^. However, whether NRF2 and 18S rRNA are mutually regulated remains unclear. Regarding ROS, nuclear mitotic apparatus protein (NuMA) is involved in cellular events such as DNA damage response, apoptosis, and P53-mediated growth arrest. In breast cancer cells, NuMA bound to 18S and 28S rRNAs and localized to rDNA promoter regions. Downregulation of NuMA expression triggered nucleolar oxidative stress and decreased pre-rRNA synthesis^169^. Furthermore, in leukemia HL-60 cells treated with iron chelator deferoxamine, rRNA synthesis in nucleoli was inhibited^170^. In conclusion, interaction between rRNAs and ferroptosis has not been completely uncovered. Role of ribosomes as the place in which proteins related to ferroptosis are synthesized may provide clues for further studies.

### PiRNAs, snRNAs, and snoRNAs

PiRNAs are the class of small ncRNA molecules distinct from miRNAs in that they are larger, lack sequence conservation, and are more complex^171^. PiRNAs are involved in tumorigenesis of variety cancers^172^. However, studies on piRNAs and ferroptosis are few. In prostate cancer, piR-31470 formed a complex with piwi-like RNA-mediated gene silencing 4 (PIWIL4). This complex recruited DNMT1, DNA methyltransferase 3 alpha, and methyl-CpG binding domain protein 2 to initiate and maintain the hypermethylation and inactivation of GSTP1. Overexpression of piR-31470 inhibited GSTP1 expression and increased vulnerability to oxidative stress and DNA damage in human prostate epithelial RWPE1 cells, resulting in tumorigenesis^173^. However, the GSTP1 inactivation may inhibit tumor growth via induction of ferroptosis once the tumors are formed. Clearly, further studies are needed to explore the roles of piRNAs in different stages of cancer. SnoRNAs are a class of small RNA molecules that mediate modifications of rRNAs, tRNAs, and snRNAs. The snoRNA ACA11 was overexpressed in multiple myeloma cells, increasing ROS and resulting in protein production and cell proliferation^174^. There are currently no reports on ferroptosis and snRNAs which mediate post-transcriptional splicing in gene expression. In cervical cancer and osteosarcoma, assembly chaperones and core proteins devoted to snRNA maturation contributed to recruiting trimethylguanosine synthase 1 to selenoprotein mRNAs including GPX1 for cap hypermethylation^175^. Future studies should focus on the possible regulation of snRNAs towards GPX families. In sum, further studies are needed to explore functions of circRNAs, tRNA, rRNAs, piRNAs, snoRNAs and snRNAs in ferroptosis. Furthermore, the network of factors modulating ferroptosis remains to be established. As ferroptosis is a process of dynamic equilibrium, any alteration of the associated factors may intersect with others. For example, GSH maintains the cytosolic labile iron pool via formation of iron-GSH complexes^176^. In addition, GSH regulates iron trafficking, and inhibition of GSH synthesis leads to diminished iron efflux following nitric oxide exposure^177^. Moreover, iron is exported via multidrug resistant protein 1 (MRP1), a known transporter of GSH conjugates^178^. GSH depletion, MRP1 inhibition or MRP1 knock-out leads to decreased iron release upon nitric oxide treatment^179^. Conversely, the secondary increase in ROS induced by iron stimulates GSH production, indicating that iron and GSH are interconnected^46^. Moreover, targets of NRF2 play a critical role in mediating iron/heme metabolism. Both FTL and FTH, the key iron storage protein, as well as FPN, which is responsible for cellular iron efflux, are controlled by NRF2^180,181^. In addition, a number of integral GSH synthesis and metabolism related enzymes including both the catalytic and modulatory subunits of GCLC, GCLM, GSS, and SLC7A11, are under the control of NRF2^182–184^. In total, regulation of ferroptosis are linked together, modulation of GSH, iron and NRF2 by ncRNAs may result in further change of each other, and finally alter ferroptosis process.

### Clinical application potential of ncRNA-associated ferroptosis

Targeting ncRNAs in cancer has yielded some promising results, however, application of ferroptosis via an ncRNA-dependent manner in clinic is facing obstacles. Inadequate understanding of specific mechanisms results in the limited use of ncRNA modifiers in ferroptosis. Furthermore, cell death occurs in a variety of ways, and numerous ncRNAs may be simultaneously regulated, thus how to ensure that the alteration of associated ncRNAs leads to ferroptosis is another problem. Moreover, ncRNAs act in various ways that may intersect with ferroptosis. For example, ferroptosis inducer *miR-210* and *H19* could modulate autophagy via targeting BECN1, ATG7, SIRT1, and HIF-1α^185–188^. In addition, *miR-146a* could regulat ROS modulator catalase and SOD2 which repressed mitochondrial function^189,190^. Alteration of autophagy or mitochondrial function resulted in multiple pathologic changes such as neuroinflammation, neurodegeneration, vessel remodeling and myocardial fibrosis, thus how to overcome these possible complications should be considered^191–194^. In addition, some pathways such as the KEAP1-NRF2 axis, is inhibited by multiple miRNAs and lncRNAs and promotes ferroptosis. Nevertheless, the repression of KEAP1-NRF2 results in the defect in cleaning of ROS and leads to susceptibility to DNA damage and tumorigenesis^195,196^. To solve these problems, future studies should address the following points. First, more ncRNAs should be identified. A ferroptosis-associated ncRNA screening platform should be established to identify the spectrum of ferroptosis associated ncRNAs and those specific to certain cancers. Second, more intensive studies using complex molecular biological experiments, such as chromosome immunoprecipitation, RNA immunoprecipitation, RNA pull-down, luciferase assays, and RNA truncation should be performed to explore the precise roles of ncRNAs in ferroptosis. Third, in order to translate fundamental experimental results into clinic, functions of ncRNAs in ferroptosis should be tested in animal models. Transgenic mouse models should be established to verify the function of ncRNAs more clearly. Fourth, in order to ensure whether ferroptosis is modulated by ncRNAs, accurate detection of ROS and iron levels, and observation of mitochondrial morphology in tumor tissues are needed. Furthermore, primary culture of tumor cells from patients should be performed to explore whether the proliferation of cancer cells is enhanced by Fer-1, which is the specific inhibitor of ferroptosis. The involvement of ncRNAs in ferroptosis in cancer can be verified in knockdown or overexpression studies. Finally, since ferroptosis occurs in not only tumors but also normal tissues, and as above, ferroptosis regulation by ncRNAs may activate other biological processes and even increase the susceptibility to tumorigenesis. Thus, both ferroptosis-related ncRNAs and associated markers of cell death, senescence, and remodeling should be assessed in patients who are suitable for ferroptosis-associated therapy. In addition, adverse events, dose-limiting toxicities and therapeutic effects should be carefully monitored through rigorous detection of organ functions, imaging of vital organs and tumors, and hematological changes during the application of ferroptosis inducers in clinic. After all, as cancer is a developmental process, the collaboration between multidisciplinary teams should be made to obtain rational therapy regimens to enhance therapeutic effect and alleviate complications.

### Conclusions and perspectives

Cancer cells may be intrinsically insensitive or evolve and develop resistance to apoptosis, resulting in cancer progression^197^. Under the development of molecular biological technologies, identification of new targets or methods to eliminate cancer cells has attracted substantial attention. Ferroptosis is a recently recognized form of programmed cell death that relies on excess intracellular ROS and consequent lipid peroxidation^198^. Ferroptosis has been successfully applied to limit tumor growth and overcome the resistance of cancer cells to apoptosis, indicating that it may be useful as a new therapeutic approach^3^. Nevertheless, the application of ferroptosis inducers in cancer therapy is limited, mainly because the specific mechanisms underlying ferroptosis remain unexplored.

NcRNAs have been proved to regulate gene expression by various manners. Numerous ncRNAs have been found to regulate behaviors of cancer cells. In recent years, researchers have examined some ferroptosis-associated ncRNAs in cancer cells. Nevertheless, the specific regulatory mechanisms have not been explored. Therefore, wider and deeper studies to explore the function of ncRNAs in ferroptosis are needed. In this review, the landscape of ncRNAs associated with ferroptosis in cancer thus far is summarized. In addition, possible obstacles during application of ncRNA-associated ferroptosis in clinic are put forward and associated solutions are suggested. However, the information summarized in this review is not sufficient to support the application of ferroptosis inducers in cancer, more ncRNAs should be identified and deeper researches should be performed. In conclusion, ncRNAs may become markers to filter cancer patients who are fit for ferroptosis therapy and become therapeutic targets of ferroptosis inducers.

## Supplementary information

Supplementary Table 1

Supplementary Table 2

## References

[1] Su, Y. L. et al. Myeloid cell-targeted miR-146a mimic inhibits NF-kB-driven inflammation and leukemia progression in vivo. *Blood*10.1182/blood.2019002045 (2019). 31805184 10.1182/blood.2019002045 PMC6966933

[2] Kim, J. et al. Structure and drug resistance of the Plasmodium falciparum transporter PfCRT. *Nature*10.1038/s41586-019-1795-x (2019). 31776516 10.1038/s41586-019-1795-x PMC6911266

[3] Rios-Luci, C. et al. Adaptive resistance to trastuzumab impairs response to neratinib and lapatinib through deregulation of cell death mechanisms. *Cancer Lett.*10.1016/j.canlet.2019.11.026 (2019). 31765734 10.1016/j.canlet.2019.11.026

[4] Dixon, S. J. et al. Ferroptosis: an iron-dependent form of nonapoptotic cell death. *Cell* **149**, 1060–1072 (2012). 22632970 10.1016/j.cell.2012.03.042 PMC3367386

[5] Gagliardi, M. et al. Aldo-keto reductases protect metastatic melanoma from ER stress-independent ferroptosis. *Cell Death Dis.* **10**, 902 (2019). 31780644 10.1038/s41419-019-2143-7 PMC6883066

[6] Bibli, S. I. et al. Shear stress regulates cystathionine gamma lyase expression to preserve endothelial redox balance and reduce membrane lipid peroxidation. *Redox Biol.* **28**, 101379 (2019). 31759247 10.1016/j.redox.2019.101379 PMC6880097

[7] Wei, S. et al. Arsenic induces pancreatic dysfunction and ferroptosis via mitochondrial ROS-autophagy-lysosomal pathway. *J. Hazard. Mater.*10.1016/j.jhazmat.2019.121390 (2019). 10.1016/j.jhazmat.2019.121390 31735470

[8] Koppula, P., Zhang, Y., Zhuang, L. & Gan, B. Amino acid transporter SLC7A11/xCT at the crossroads of regulating redox homeostasis and nutrient dependency of cancer. *Cancer Commun.* **38**, 12 (2018). 10.1186/s40880-018-0288-x PMC5993148 29764521

[9] Lang, X. et al. Radiotherapy and immunotherapy promote tumoral lipid oxidation and ferroptosis via synergistic repression of SLC7A11. *Cancer Discov.*10.1158/2159-8290.CD-19-0338 (2019). 31554642 10.1158/2159-8290.CD-19-0338 PMC6891128

[10] Muri, J., Thut, H., Bornkamm, G. W. & Kopf, M. B1 and marginal zone B cellsr but not follicular B2 cells Require Gpx4 to prevent lipid peroxidation and ferroptosis. *Cell Rep.* **29**, 2731–2744 e2734 (2019). 31775041 10.1016/j.celrep.2019.10.070

[11] Xie, Y. et al. Ferroptosis: process and function. *Cell Death Differ.* **23**, 369–379 (2016). 26794443 10.1038/cdd.2015.158 PMC5072448

[12] Doll, S. et al. FSP1 is a glutathione-independent ferroptosis suppressor. *Nature* **575**, 693–698 (2019). 31634899 10.1038/s41586-019-1707-0

[13] Kajarabille, N. & Latunde-Dada, G. O. Programmed cell-death by ferroptosis: antioxidants as mitigators. *Int. J. Mol. Sci.*10.3390/ijms20194968 (2019). 10.3390/ijms20194968 PMC6801403 31597407

[14] Hassannia, B., Vandenabeele, P. & Vanden Berghe, T. Targeting ferroptosis to iron out cancer. *Cancer Cell* **35**, 830–849 (2019). 31105042 10.1016/j.ccell.2019.04.002

[15] Sui, X. et al. RSL3 drives ferroptosis through GPX4 inactivation and ROS production in colorectal cancer. *Front. Pharmacol.* **9**, 1371 (2018). 30524291 10.3389/fphar.2018.01371 PMC6262051

[16] Tang, H. et al. Dual GSH-exhausting sorafenib loaded manganese-silica nanodrugs for inducing the ferroptosis of hepatocellular carcinoma cells. *Int. J. Pharm.* **572**, 118782 (2019). 31678528 10.1016/j.ijpharm.2019.118782

[17] Yu, Y. et al. The ferroptosis inducer erastin enhances sensitivity of acute myeloid leukemia cells to chemotherapeutic agents. *Mol. Cell. Oncol.* **2**, e1054549 (2015). 27308510 10.1080/23723556.2015.1054549 PMC4905356

[18] Roh, J. L., Kim, E. H., Jang, H. J., Park, J. Y. & Shin, D. Induction of ferroptotic cell death for overcoming cisplatin resistance of head and neck cancer. *Cancer Lett.* **381**, 96–103 (2016). 27477897 10.1016/j.canlet.2016.07.035

[19] Moreau, C. et al. Iron as a therapeutic target for Parkinson’s disease. *Mov. Disord.* **33**, 568–574 (2018). 29380903 10.1002/mds.27275

[20] Su, L. et al. Pannexin 1 mediates ferroptosis that contributes to renal ischemia/reperfusion injury. *J. Biol. Chem.*10.1074/jbc.RA119.010949 (2019). 31694915 10.1074/jbc.RA119.010949 PMC6916502

[21] Di Bella, S. et al. A benchmarking of pipelines for detecting ncRNAs from RNA-Seq data. *Brief. Bioinform.*10.1093/bib/bbz110 (2019). 10.1093/bib/bbz110 31740918

[22] Alzhrani, R. et al. Improving the therapeutic efficiency of noncoding RNAs in cancers using targeted drug delivery systems. *Drug Discov. Today*10.1016/j.drudis.2019.11.006 (2019). 31758914 10.1016/j.drudis.2019.11.006 PMC7200292

[23] Wang, J. et al. ncRNA-encoded peptides or proteins and cancer. *Mol. Ther.* **27**, 1718–1725 (2019). 31526596 10.1016/j.ymthe.2019.09.001 PMC6822234

[24] Jusic, A., Devaux, Y. & Action, E. U.-C. C. Noncoding RNAs in Hypertension. *Hypertension* **74**, 477–492 (2019). 31352819 10.1161/HYPERTENSIONAHA.119.13412 PMC6686966

[25] Yao, R. W., Wang, Y. & Chen, L. L. Cellular functions of long noncoding RNAs. *Nat. Cell Biol.* **21**, 542–551 (2019). 31048766 10.1038/s41556-019-0311-8

[26] Costa, V. et al. MiR-675-5p supports hypoxia induced epithelial to mesenchymal transition in colon cancer cells. *Oncotarget* **8**, 24292–24302 (2017). 28061476 10.18632/oncotarget.14464 PMC5421847

[27] Pawlowska, E., Szczepanska, J. & Blasiak, J. The long noncoding RNA HOTAIR in breast cancer: does autophagy play a role? *Int. J. Mol. Sci.*10.3390/ijms18112317 (2017). 10.3390/ijms18112317 PMC5713286 29469819

[28] Kong, Z. et al. Circular RNA circFOXO3 promotes prostate cancer progression through sponging miR-29a-3p. *J. Cell. Mol. Med.*10.1111/jcmm.14791 (2019). 31733095 10.1111/jcmm.14791 PMC6933405

[29] Majidinia, M., Karimian, A., Alemi, F., Yousefi, B. & Safa, A. Targeting miRNAs by polyphenols: novel therapeutic strategy for aging. *Biochem. Pharmacol.*10.1016/j.bcp.2019.113688 (2019). 10.1016/j.bcp.2019.113688 31682793

[30] Tomita, K. et al. MiR-7-5p is a key factor that controls radioresistance via intracellular Fe(2+) content in clinically relevant radioresistant cells. *Biochem. Biophys. Res. Commun.* **518**, 712–718 (2019). 31472959 10.1016/j.bbrc.2019.08.117

[31] Zhang, K. et al. miR-9 regulates ferroptosis by targeting glutamic-oxaloacetic transaminase GOT1 in melanoma. *Mol. Carcinog.* **57**, 1566–1576 (2018). 30035324 10.1002/mc.22878

[32] Wang, M. et al. Long noncoding RNA LINC00336 inhibits ferroptosis in lung cancer by functioning as a competing endogenous RNA. *Cell Death Differ.* **26**, 2329–2343 (2019). 30787392 10.1038/s41418-019-0304-y PMC6889193

[33] Hsu, J. L. et al. Glutathione peroxidase 8 negatively regulates caspase-4/11 to protect against colitis. *EMBO Mol. Med.*10.15252/emmm.201809386 (2019). 10.15252/emmm.201809386 PMC6949489 31782617

[34] Koeberle, S. C. et al. Distinct and overlapping functions of glutathione peroxidases 1 and 2 in limiting NF-kappaB-driven inflammation through redox-active mechanisms. *Redox Biol.* **28**, 101388 (2019). 31765890 10.1016/j.redox.2019.101388 PMC6883322

[35] Desideri, E., Ciccarone, F. & Ciriolo, M. R. Targeting glutathione metabolism: partner in crime in anticancer therapy. *Nutrients*10.3390/nu11081926 (2019). 10.3390/nu11081926 PMC6724225 31426306

[36] Nunes, S. C. & Serpa, J. Glutathione in ovarian cancer: a double-edged sword. *Int. J. Mol. Sci.*10.3390/ijms19071882 (2018). 10.3390/ijms19071882 PMC6073569 29949936

[37] Anderton, B. et al. MYC-driven inhibition of the glutamate-cysteine ligase promotes glutathione depletion in liver cancer. *EMBO Rep.* **18**, 569–585 (2017). 28219903 10.15252/embr.201643068 PMC5376764

[38] Li, P. et al. MicroRNA-218 increases the sensitivity of bladder cancer to cisplatin by targeting Glut1. *Cell. Physiol. Biochem.* **41**, 921–932 (2017). 28222430 10.1159/000460505

[39] Huang, J., Wang, Y., Guo, Y. & Sun, S. Down-regulated microRNA-152 induces aberrant DNA methylation in hepatitis B virus-related hepatocellular carcinoma by targeting DNA methyltransferase 1. *Hepatology* **52**, 60–70 (2010). 20578129 10.1002/hep.23660

[40] Lv, L., An, X., Li, H. & Ma, L. Effect of miR-155 knockdown on the reversal of doxorubicin resistance in human lung cancer A549/dox cells. *Oncol. Lett.* **11**, 1161–1166 (2016). 26893712 10.3892/ol.2015.3995 PMC4734087

[41] Kefas, B. et al. Pyruvate kinase M2 is a target of the tumor-suppressive microRNA-326 and regulates the survival of glioma cells. *NeuroOncology* **12**, 1102–1112 (2010). 10.1093/neuonc/noq080 PMC3098027 20667897

[42] Drayton, R. M. et al. Reduced expression of miRNA-27a modulates cisplatin resistance in bladder cancer by targeting the cystine/glutamate exchanger SLC7A11. *Clin. Cancer Res.* **20**, 1990–2000 (2014). 24516043 10.1158/1078-0432.CCR-13-2805 PMC3974662

[43] Pathi, S. S. et al. GT-094, a NO-NSAID, inhibits colon cancer cell growth by activation of a reactive oxygen species-microRNA-27a: ZBTB10-specificity protein pathway. *Mol. Cancer Res.* **9**, 195–202 (2011). 21156786 10.1158/1541-7786.MCR-10-0363 PMC3069691

[44] Dong, Z. et al. Effect of microRNA-21 on multidrug resistance reversal in A549/DDP human lung cancer cells. *Mol. Med. Rep.* **11**, 682–690 (2015). 25323306 10.3892/mmr.2014.2662

[45] He, H. W., Wang, N. N., Yi, X. M., Tang, C. P. & Wang, D. Low-level serum miR-24-2 is associated with the progression of colorectal cancer. *Cancer Biomark.* **21**, 261–267 (2018). 29171985 10.3233/CBM-170321 PMC13078269

[46] Yang, H. et al. MicroRNA-497 regulates cisplatin chemosensitivity of cervical cancer by targeting transketolase. *Am. J. Cancer Res.* **6**, 2690–2699 (2016). 27904781 PMC5126283

[47] Wang, D. et al. Role and mechanisms of microRNA503 in drug resistance reversal in HepG2/ADM human hepatocellular carcinoma cells. *Mol. Med. Rep.* **10**, 3268–3274 (2014). 25269574 10.3892/mmr.2014.2591

[48] Zhu, J. et al. Knockdown of long non-coding RNA XIST inhibited doxorubicin resistance in colorectal cancer by upregulation of miR-124 and downregulation of SGK1. *Cell. Physiol. Biochem.* **51**, 113–128 (2018). 30439718 10.1159/000495168

[49] Ma, H. et al. Identification of reciprocal microRNA-mRNA pairs associated with metastatic potential disparities in human prostate cancer cells and signaling pathway analysis. *J. Cell. Biochem.* **120**, 17779–17790 (2019). 31127646 10.1002/jcb.29045

[50] Ghanbarian, M., Afgar, A., Yadegarazari, R., Najafi, R. & Teimoori-Toolabi, L. Through oxaliplatin resistance induction in colorectal cancer cells, increasing ABCB1 level accompanies decreasing level of miR-302c-5p, miR-3664-5p and miR-129-5p. *Biomed. Pharmacother.* **108**, 1070–1080 (2018). 30372807 10.1016/j.biopha.2018.09.112

[51] Singh, S., Shukla, G. C. & Gupta, S. MicroRNA regulating glutathione S-transferase P1 in prostate cancer. *Curr. Pharmacol. Rep.* **1**, 79–88 (2015). 25774339 10.1007/s40495-014-0009-3 PMC4354897

[52] Uchida, Y. et al. MiR-133a induces apoptosis through direct regulation of GSTP1 in bladder cancer cell lines. *Urol Oncol.* **31**, 115–123 (2013). 21396852 10.1016/j.urolonc.2010.09.017

[53] Moriya, Y. et al. Tumor suppressive microRNA-133a regulates novel molecular networks in lung squamous cell carcinoma. *J. Hum. Genet.* **57**, 38–45 (2012). 22089643 10.1038/jhg.2011.126

[54] Lin, C., Xie, L., Lu, Y., Hu, Z. & Chang, J. miR-133b reverses cisplatin resistance by targeting GSTP1 in cisplatin-resistant lung cancer cells. *Int. J. Mol. Med.* **41**, 2050–2058 (2018). 29328427 10.3892/ijmm.2018.3382 PMC5810210

[55] Xue, J. et al. The hsa-miR-181a-5p reduces oxidation resistance by controlling SECISBP2 in osteoarthritis. *BMC Musculoskelet. Disord.* **19**, 355 (2018). 30286747 10.1186/s12891-018-2273-6 PMC6172777

[56] Xu, Y. et al. miR-17* suppresses tumorigenicity of prostate cancer by inhibiting mitochondrial antioxidant enzymes. *PLoS ONE* **5**, e14356 (2010). 21203553 10.1371/journal.pone.0014356 PMC3008681

[57] Xu, Z. et al. miR-17-3p downregulates mitochondrial antioxidant enzymes and enhances the radiosensitivity of prostate cancer cells. *Mol. Ther. Nucleic Acids* **13**, 64–77 (2018). 30240971 10.1016/j.omtn.2018.08.009 PMC6143750

[58] Liu, Q., Bai, W., Huang, F., Tang, J. & Lin, X. Downregulation of microRNA-196a inhibits stem cell self-renewal ability and stemness in non-small-cell lung cancer through upregulating GPX3 expression. *Int. J. Biochem. Cell Biol.* **115**, 105571 (2019). 31352088 10.1016/j.biocel.2019.105571

[59] Choi, J. Y., An, B. C., Jung, I. J., Kim, J. H. & Lee, S. W. MiR-921 directly downregulates GPx3 in A549 lung cancer cells. *Gene* **700**, 163–167 (2019). 30898707 10.1016/j.gene.2019.02.086

[60] Arefieva Olga, D., Vasilyeva Marina, S., Zemnukhova Liudmila, A. & Timochkina Anna, S. Heterogeneous photo-Fenton oxidation of lignin of rice husk alkaline hydrolysates using Fe-impregnated silica catalysts. *Environ. Technol.*10.1080/09593330.2019.1697376 (2019). 10.1080/09593330.2019.1697376 31762392

[61] Abeyawardhane, D. L. & Lucas, H. R. Iron redox chemistry and implications in the Parkinson’s disease brain. *Oxid. Med. Cell. Longev.* **2019**, 4609702 (2019). 31687080 10.1155/2019/4609702 PMC6803728

[62] Wang, F. et al. Iron and leukemia: new insights for future treatments. *J. Exp. Clin. Cancer Res.* **38**, 406 (2019). 31519186 10.1186/s13046-019-1397-3 PMC6743129

[63] Hamara, K. et al. Alterations in expression profile of iron-related genes in colorectal cancer. *Mol. Biol. Rep.* **40**, 5573–5585 (2013). 24078156 10.1007/s11033-013-2659-3 PMC3824343

[64] Kindrat, I. et al. MicroRNA-152-mediated dysregulation of hepatic transferrin receptor 1 in liver carcinogenesis. *Oncotarget* **7**, 1276–1287 (2016). 26657500 10.18632/oncotarget.6004 PMC4811459

[65] Greene, C. M., Varley, R. B. & Lawless, M. W. MicroRNAs and liver cancer associated with iron overload: therapeutic targets unravelled. *World J. Gastroenterol.* **19**, 5212–5226 (2013). 23983424 10.3748/wjg.v19.i32.5212 PMC3752555

[66] Kong, Y. et al. Ferroportin downregulation promotes cell proliferation by modulating the Nrf2-miR-17-5p axis in multiple myeloma. *Cell Death Dis.* **10**, 624 (2019). 31423010 10.1038/s41419-019-1854-0 PMC6698482

[67] Babu, K. R. & Muckenthaler, M. U. miR-20a regulates expression of the iron exporter ferroportin in lung cancer. *J. Mol. Med.* **94**, 347–359 (2016). 26560875 10.1007/s00109-015-1362-3 PMC4803811

[68] Chen, Y. et al. Myeloid zinc-finger 1 (MZF-1) suppresses prostate tumor growth through enforcing ferroportin-conducted iron egress. *Oncogene* **34**, 3839–3847 (2015). 25284586 10.1038/onc.2014.310

[69] Stuhn, L., Auernhammer, J. & Dietz, C. pH-depended protein shell dis- and reassembly of ferritin nanoparticles revealed by atomic force microscopy. *Sci. Rep.* **9**, 17755 (2019). 31780685 10.1038/s41598-019-53943-3 PMC6883049

[70] Chan, J. J. et al. A FTH1 gene:pseudogene:microRNA network regulates tumorigenesis in prostate cancer. *Nucleic Acids Res.* **46**, 1998–2011 (2018). 29240947 10.1093/nar/gkx1248 PMC5829750

[71] Shpyleva, S. I. et al. Role of ferritin alterations in human breast cancer cells. *Breast Cancer Res. Treat.* **126**, 63–71 (2011). 20390345 10.1007/s10549-010-0849-4

[72] Chekhun, V. F. et al. Iron metabolism disturbances in the MCF-7 human breast cancer cells with acquired resistance to doxorubicin and cisplatin. *Int. J. Oncol.* **43**, 1481–1486 (2013). 23969999 10.3892/ijo.2013.2063

[73] Yoshioka, Y., Kosaka, N., Ochiya, T. & Kato, T. Micromanaging iron homeostasis: hypoxia-inducible micro-RNA-210 suppresses iron homeostasis-related proteins. *J. Biol. Chem.* **287**, 34110–34119 (2012). 22896707 10.1074/jbc.M112.356717 PMC3464520

[74] Gee, H. E., Ivan, C., Calin, G. A. & Ivan, M. HypoxamiRs and cancer: from biology to targeted therapy. *Antioxid. Redox Signal.* **21**, 1220–1238 (2014). 24111776 10.1089/ars.2013.5639 PMC4142802

[75] Zou, C. et al. Heme oxygenase-1 retards hepatocellular carcinoma progression through the microRNA pathway. *Oncol. Rep.* **36**, 2715–2722 (2016). 27571925 10.3892/or.2016.5056

[76] Lobello, N. et al. Ferritin heavy chain is a negative regulator of ovarian cancer stem cell expansion and epithelial to mesenchymal transition. *Oncotarget* **7**, 62019–62033 (2016). 27566559 10.18632/oncotarget.11495 PMC5308708

[77] Sriramoju, B., Kanwar, R. K. & Kanwar, J. R. Lactoferrin induced neuronal differentiation: a boon for brain tumours. *Int. J. Dev. Neurosci.* **41**, 28–36 (2015). 25498991 10.1016/j.ijdevneu.2014.12.005

[78] Cloer, E. W., Goldfarb, D., Schrank, T. P., Weissman, B. E. & Major, M. B. NRF2 activation in cancer: from DNA to protein. *Cancer Res.* **79**, 889–898 (2019). 30760522 10.1158/0008-5472.CAN-18-2723 PMC6397706

[79] Gai, C. et al. Acetaminophen sensitizing erastin-induced ferroptosis via modulation of Nrf2/heme oxygenase-1 signaling pathway in non-small-cell lung cancer. *J. Cell. Physiol.*10.1002/jcp.29221 (2019). 31541463 10.1002/jcp.29221 PMC13482668

[80] Yamamoto, S. et al. The impact of miRNA-based molecular diagnostics and treatment of NRF2-stabilized tumors. *Mol. Cancer Res.* **12**, 58–68 (2014). 24307696 10.1158/1541-7786.MCR-13-0246-T

[81] Tian, Y. et al. Emerging roles of Nrf2 signal in non-small cell lung cancer. *J. Hematol. Oncol.* **9**, 14 (2016). 26922479 10.1186/s13045-016-0246-5 PMC4769825

[82] Wang, B., Teng, Y. & Liu, Q. MicroRNA-153 regulates NRF2 expression and is associated with breast carcinogenesis. *Clin. Lab.* **62**, 39–47 (2016). 27012032 10.7754/clin.lab.2015.150518

[83] Qaisiya, M., Coda Zabetta, C. D., Bellarosa, C. & Tiribelli, C. Bilirubin mediated oxidative stress involves antioxidant response activation via Nrf2 pathway. *Cell. Signal.* **26**, 512–520 (2014). 24308969 10.1016/j.cellsig.2013.11.029

[84] Narasimhan, M. et al. Identification of novel microRNAs in post-transcriptional control of Nrf2 expression and redox homeostasis in neuronal, SH-SY5Y cells. *PLoS ONE* **7**, e51111 (2012). 23236440 10.1371/journal.pone.0051111 PMC3517581

[85] Shi, L. et al. miR-340 reverses cisplatin resistance of hepatocellular carcinoma cell lines by targeting Nrf2-dependent antioxidant pathway. *Asian Pac. J. Cancer Prev.* **15**, 10439–10444 (2014). 25556489 10.7314/apjcp.2014.15.23.10439

[86] Hamalainen, M. et al. NRF1 and NRF2 mRNA and protein expression decrease early during melanoma carcinogenesis: an insight into survival and MicroRNAs. *Oxid. Med. Cell. Longev.* **2019**, 2647068 (2019). 31687076 10.1155/2019/2647068 PMC6794976

[87] Yin, Y. et al. miR1443p regulates the resistance of lung cancer to cisplatin by targeting Nrf2. *Oncol. Rep.* **40**, 3479–3488 (2018). 30542710 10.3892/or.2018.6772

[88] Raghunath, A., Sundarraj, K., Arfuso, F., Sethi, G. & Perumal, E. Dysregulation of Nrf2 in hepatocellular carcinoma: role in cancer progression and chemoresistance. *Cancers*10.3390/cancers10120481 (2018). 10.3390/cancers10120481 PMC6315366 30513925

[89] Zhou, C. et al. MicroRNA-144 modulates oxidative stress tolerance in SH-SY5Y cells by regulating nuclear factor erythroid 2-related factor 2-glutathione axis. *Neurosci. Lett.* **655**, 21–27 (2017). 28663050 10.1016/j.neulet.2017.06.045

[90] Zhou, S. et al. miR-144 reverses chemoresistance of hepatocellular carcinoma cell lines by targeting Nrf2-dependent antioxidant pathway. *Am. J. Transl. Res.* **8**, 2992–3002 (2016). 27508019 PMC4969435

[91] Wu, L. et al. lncRNA KRAL reverses 5-fluorouracil resistance in hepatocellular carcinoma cells by acting as a ceRNA against miR-141. *Cell Commun. Signal.* **16**, 47 (2018). 30119680 10.1186/s12964-018-0260-z PMC6098660

[92] Fabrizio, F. P., Sparaneo, A., Trombetta, D. & Muscarella, L. A. Epigenetic versus genetic deregulation of the KEAP1/NRF2 axis in solid tumors: focus on methylation and noncoding RNAs. *Oxid. Med. Cell. Longev.* **2018**, 2492063 (2018). 29643973 10.1155/2018/2492063 PMC5872633

[93] Shi, L. et al. MiR-141 activates Nrf2-dependent antioxidant pathway via down-regulating the expression of Keap1 conferring the resistance of hepatocellular carcinoma cells to 5-fluorouracil. *Cell. Physiol. Biochem.* **35**, 2333–2348 (2015). 25896253 10.1159/000374036

[94] Khan, A. U. H. et al. Human leukemic cells performing oxidative phosphorylation (OXPHOS) generate an antioxidant response independently of reactive oxygen species (ROS) production. *EBioMedicine* **3**, 43–53 (2016). 26870816 10.1016/j.ebiom.2015.11.045 PMC4739420

[95] Akdemir, B., Nakajima, Y., Inazawa, J. & Inoue, J. miR-432 induces NRF2 stabilization by directly targeting KEAP1. *Mol. Cancer Res.* **15**, 1570–1578 (2017). 28760781 10.1158/1541-7786.MCR-17-0232

[96] Liu, M. et al. Methylseleninic acid activates Keap1/Nrf2 pathway via up-regulating miR-200a in human oesophageal squamous cell carcinoma cells. *Biosci. Rep.*10.1042/BSR20150092 (2015). 10.1042/BSR20150092 PMC4613709 26341629

[97] Karihtala, P. et al. Expression levels of microRNAs miR-93 and miR-200a in pancreatic adenocarcinoma with special reference to differentiation and relapse-free survival. *Oncology* **96**, 164–170 (2019). 30537722 10.1159/000494274

[98] Shah, N. M., Zaitseva, L., Bowles, K. M., MacEwan, D. J. & Rushworth, S. A. NRF2-driven miR-125B1 and miR-29B1 transcriptional regulation controls a novel anti-apoptotic miRNA regulatory network for AML survival. *Cell Death Differ.* **22**, 654–664 (2015). 25323587 10.1038/cdd.2014.152 PMC4356334

[99] Chen, Y. F. et al. miR-125b suppresses oral oncogenicity by targeting the anti-oxidative gene PRXL2A. *Redox Biol.* **22**, 101140 (2019). 30785086 10.1016/j.redox.2019.101140 PMC6383183

[100] Sun, W. et al. Nrf2-miR-129-3p-mTOR axis controls an miRNA regulatory network involved in HDACi-induced autophagy. *Mol. Ther.* **27**, 1039–1050 (2019). 30852137 10.1016/j.ymthe.2019.02.010 PMC6520288

[101] Cai, Z. et al. Nrf2-regulated miR-380-3p blocks the translation of Sp3 protein and its mediation of paraquat-induced toxicity in mouse neuroblastoma N2a cells. *Toxicol. Sci.*10.1093/toxsci/kfz162 (2019). 31368498 10.1093/toxsci/kfz162 PMC6760285

[102] Jung, K. A., Lee, S. & Kwak, M. K. NFE2L2/NRF2 activity is linked to mitochondria and AMP-activated protein kinase signaling in cancers through miR-181c/Mitochondria-encoded cytochrome c oxidase regulation. *Antioxid. Redox Signal.* **27**, 945–961 (2017). 28383996 10.1089/ars.2016.6797

[103] Tertil, M. et al. Nrf2-heme oxygenase-1 axis in mucoepidermoid carcinoma of the lung: antitumoral effects associated with down-regulation of matrix metalloproteinases. *Free Radic. Biol. Med.* **89**, 147–157 (2015). 26393425 10.1016/j.freeradbiomed.2015.08.004

[104] Aydin, Y. et al. Hepatic stress response in HCV infection promotes STAT3-mediated inhibition of HNF4A-miR-122 feedback loop in liver fibrosis and cancer progression. *Cancers*10.3390/cancers11101407 (2019). 10.3390/cancers11101407 PMC6827087 31547152

[105] Martino, T. et al. The orally active pterocarpanquinone LQB-118 exhibits cytotoxicity in prostate cancer cell and tumor models through cellular redox stress. *Prostate* **78**, 140–151 (2018). 29105806 10.1002/pros.23455 PMC5726914

[106] Choi, B. H., Ryu, D. Y., Ryoo, I. G. & Kwak, M. K. NFE2L2/NRF2 silencing-inducible miR-206 targets c-MET/EGFR and suppresses BCRP/ABCG2 in cancer cells. *Oncotarget* **8**, 107188–107205 (2017). 29291022 10.18632/oncotarget.22513 PMC5739807

[107] Singh, A. et al. Transcription factor NRF2 regulates miR-1 and miR-206 to drive tumorigenesis. *J. Clin. Investig.* **123**, 2921–2934 (2013). 23921124 10.1172/JCI66353 PMC3696551

[108] Griess, B., Tom, E., Domann, F. & Teoh-Fitzgerald, M. Extracellular superoxide dismutase and its role in cancer. *Free Radic. Biol. Med.* **112**, 464–479 (2017). 28842347 10.1016/j.freeradbiomed.2017.08.013 PMC5685559

[109] Lu, Z. et al. MicroRNA-21 promotes cell transformation by targeting the programmed cell death 4 gene. *Oncogene* **27**, 4373–4379 (2008). 18372920 10.1038/onc.2008.72 PMC11968769

[110] Jiang, Y. et al. The role of TGF-beta1-miR-21-ROS pathway in bystander responses induced by irradiated non-small-cell lung cancer cells. *Br. J. Cancer* **111**, 772–780 (2014). 24992582 10.1038/bjc.2014.368 PMC4134503

[111] Tu, H. et al. Oxidative stress upregulates PDCD4 expression in patients with gastric cancer via miR-21. *Curr. Pharm. Des.* **20**, 1917–1923 (2014). 23888942 10.2174/13816128113199990547

[112] Pratheeshkumar, P. et al. Oncogenic transformation of human lung bronchial epithelial cells induced by arsenic involves ROS-dependent activation of STAT3-miR-21-PDCD4 mechanism. *Sci. Rep.* **6**, 37227 (2016). 27876813 10.1038/srep37227 PMC5120334

[113] Zhang, X. et al. MicroRNA-21 modulates the levels of reactive oxygen species by targeting SOD3 and TNFalpha. *Cancer Res.* **72**, 4707–4713 (2012). 22836756 10.1158/0008-5472.CAN-12-0639 PMC3445705

[114] Su, Y. et al. Silencing miR-21 induces polarization of astrocytes to the A2 phenotype and improves the formation of synapses by targeting glypican 6 via the signal transducer and activator of transcription-3 pathway after acute ischemic spinal cord injury. *FASEB J.* **33**, 10859–10871 (2019). 31266356 10.1096/fj.201900743R

[115] Adam, O. et al. Role of miR-21 in the pathogenesis of atrial fibrosis. *Basic Res. Cardiol.* **107**, 278 (2012). 22760500 10.1007/s00395-012-0278-0

[116] Galuppini, F. et al. Programmed cell death 4 (PDCD4) as a novel prognostic marker for papillary thyroid carcinoma. *Cancer Manage. Res.* **11**, 7845–7855 (2019). 10.2147/CMAR.S194344 PMC6708393 31692513

[117] Cui, Y., She, K., Tian, D., Zhang, P. & Xin, X. miR-146a inhibits proliferation and enhances chemosensitivity in epithelial ovarian cancer via reduction of SOD2. *Oncol. Res.* **23**, 275–282 (2016). 27131313 10.3727/096504016X14562725373798 PMC7838621

[118] Wang, Q. et al. Receptor-interacting protein 1 increases chemoresistance by maintaining inhibitor of apoptosis protein levels and reducing reactive oxygen species through a microRNA-146a-mediated catalase pathway. *J. Biol. Chem.* **289**, 5654–5663 (2014). 24425875 10.1074/jbc.M113.526152 PMC3937640

[119] Shin, B. et al. miR526b and miR655 induce oxidative stress in breast cancer. *Int. J. Mol. Sci.*10.3390/ijms20164039 (2019). 10.3390/ijms20164039 PMC6720387 31430859

[120] Hao, C. et al. MicroRNA-124 regulates the radiosensitivity of non-small cell lung cancer cells by targeting TXNRD1. *Oncol. Lett.* **13**, 2071–2078 (2017). 28454363 10.3892/ol.2017.5701 PMC5403322

[121] Gomes, S. E. et al. Convergence of miR-143 overexpression, oxidative stress and cell death in HCT116 human colon cancer cells. *PLoS ONE* **13**, e0191607 (2018). 29360852 10.1371/journal.pone.0191607 PMC5779689

[122] Sun, M. et al. MiR-99a regulates ROS-mediated invasion and migration of lung adenocarcinoma cells by targeting NOX4. *Oncol. Rep.* **35**, 2755–2766 (2016). 26986073 10.3892/or.2016.4672

[123] Keklikoglou, I. et al. MicroRNA-520/373 family functions as a tumor suppressor in estrogen receptor negative breast cancer by targeting NF-kappaB and TGF-beta signaling pathways. *Oncogene* **31**, 4150–4163 (2012). 22158050 10.1038/onc.2011.571

[124] Bott, A. et al. miRNA-1246 induces pro-inflammatory responses in mesenchymal stem/stromal cells by regulating PKA and PP2A. *Oncotarget* **8**, 43897–43914 (2017). 28159925 10.18632/oncotarget.14915 PMC5546423

[125] Sun, J. et al. miR-137 mediates the functional link between c-Myc and EZH2 that regulates cisplatin resistance in ovarian cancer. *Oncogene* **38**, 564–580 (2019). 30166592 10.1038/s41388-018-0459-x PMC7474467

[126] Zhou, S. et al. miR199a3p/Sp1/LDHA axis controls aerobic glycolysis in testicular tumor cells. *Int. J. Mol. Med.* **42**, 2163–2174 (2018). 30015851 10.3892/ijmm.2018.3771

[127] Mazzu, Y. Z. et al. miR-193b-regulated signaling networks serve as tumor suppressors in liposarcoma and promote adipogenesis in adipose-derived stem cells. *Cancer Res.* **77**, 5728–5740 (2017). 28882999 10.1158/0008-5472.CAN-16-2253

[128] Ali, O., Darwish, H. A., Eldeib, K. M. & Abdel Azim, S. A. miR-26a potentially contributes to the regulation of fatty acid and sterol metabolism in vitro human HepG2 cell model of nonalcoholic fatty liver disease. *Oxid. Med. Cell. Longev.* **2018**, 8515343 (2018). 30402207 10.1155/2018/8515343 PMC6196797

[129] Beccafico, S. et al. Artesunate induces ROS- and p38 MAPK-mediated apoptosis and counteracts tumor growth in vivo in embryonal rhabdomyosarcoma cells. *Carcinogenesis* **36**, 1071–1083 (2015). 26153023 10.1093/carcin/bgv098

[130] Wan, Y. et al. Identification of four oxidative stress-responsive microRNAs, miR-34a-5p, miR-1915-3p, miR-638, and miR-150-3p, in hepatocellular carcinoma. *Oxid. Med. Cell. Longev.* **2017**, 5189138 (2017). 28811864 10.1155/2017/5189138 PMC5546075

[131] Ciesla, M. et al. Heme oxygenase-1 controls an HDAC4-miR-206 pathway of oxidative stress in rhabdomyosarcoma. *Cancer Res.* **76**, 5707–5718 (2016). 27488535 10.1158/0008-5472.CAN-15-1883

[132] Xu, X. et al. Oxidative stress-induced miRNAs modulate AKT signaling and promote cellular senescence in uterine leiomyoma. *J. Mol. Med.* **96**, 1095–1106 (2018). 30097674 10.1007/s00109-018-1682-1 PMC6135677

[133] Lan, J., Huang, Z., Han, J., Shao, J. & Huang, C. Redox regulation of microRNAs in cancer. *Cancer Lett.* **418**, 250–259 (2018). 29330105 10.1016/j.canlet.2018.01.010

[134] He, J. et al. Reactive oxygen species regulate ERBB2 and ERBB3 expression via miR-199a/125b and DNA methylation. *EMBO Rep.* **13**, 1116–1122 (2012). 23146892 10.1038/embor.2012.162 PMC3512405

[135] Donzelli, S. et al. Epigenetic silencing of miR-145-5p contributes to brain metastasis. *Oncotarget* **6**, 35183–35201 (2015). 26440147 10.18632/oncotarget.5930 PMC4742098

[136] Jutooru, I. et al. Mechanism of action of phenethylisothiocyanate and other reactive oxygen species-inducing anticancer agents. *Mol. Cell. Biol.* **34**, 2382–2395 (2014). 24732804 10.1128/MCB.01602-13 PMC4054319

[137] Hong, L. et al. The miR-17-92 cluster of microRNAs confers tumorigenicity by inhibiting oncogene-induced senescence. *Cancer Res.* **70**, 8547–8557 (2010). 20851997 10.1158/0008-5472.CAN-10-1938 PMC2970743

[138] Chen, T., Yu, Q., Xin, L. & Guo, L. Circular RNA circC3P1 restrains kidney cancer cell activity by regulating miR-21/PTEN axis and inactivating PI3K/AKT and NF- kB pathways. *J. Cell. Physiol.*10.1002/jcp.29296 (2019). 31643094 10.1002/jcp.29296

[139] Wu, Z. Y., Trenner, M., Boon, R. A., Spin, J. M. & Maegdefessel, L. Long noncoding RNAs in key cellular processes involved in aortic aneurysms. *Atherosclerosis* **292**, 112–118 (2019). 31785492 10.1016/j.atherosclerosis.2019.11.013 PMC6949864

[140] Mao, C. et al. A G3BP1-interacting lncRNA promotes ferroptosis and apoptosis in cancer via nuclear sequestration of p53. *Cancer Res.* **78**, 3484–3496 (2018). 29588351 10.1158/0008-5472.CAN-17-3454 PMC8073197

[141] Qi, W. et al. LncRNA GABPB1-AS1 and GABPB1 regulate oxidative stress during erastin-induced ferroptosis in HepG2 hepatocellular carcinoma cells. *Sci. Rep.* **9**, 16185 (2019). 31700067 10.1038/s41598-019-52837-8 PMC6838315

[142] Li, Y. et al. Inhibition of long non-coding RNA ROR reverses resistance to Tamoxifen by inducing autophagy in breast cancer. *Tumour Biol.* **39**, 1010428317705790 (2017). 28635401 10.1177/1010428317705790

[143] Wang, S. et al. NEAT1 paraspeckle promotes human hepatocellular carcinoma progression by strengthening IL-6/STAT3 signaling. *Oncoimmunology* **7**, e1503913 (2018). 30377567 10.1080/2162402X.2018.1503913 PMC6205018

[144] Chen, J. L. et al. Overexpression of long noncoding RNA LINC01419 in esophageal squamous cell carcinoma and its relation to the sensitivity to 5-fluorouracil by mediating GSTP1 methylation. *Therap. Adv. Med. Oncol.* **11**, 1758835919838958 (2019). 31019568 10.1177/1758835919838958 PMC6463338

[145] Zheng, Z. G. et al. The essential role of H19 contributing to cisplatin resistance by regulating glutathione metabolism in high-grade serous ovarian cancer. *Sci. Rep.* **6**, 26093 (2016). 27193186 10.1038/srep26093 PMC4872133

[146] Xu, Y. et al. Long non-coding RNA PVT1/miR-150/ HIG2 axis regulates the proliferation, invasion and the balance of iron metabolism of hepatocellular carcinoma. *Cell. Physiol. Biochem.* **49**, 1403–1419 (2018). 30205391 10.1159/000493445

[147] Di Sanzo, M. et al. shRNA targeting of ferritin heavy chain activates H19/miR-675 axis in K562 cells. *Gene* **657**, 92–99 (2018). 29544765 10.1016/j.gene.2018.03.027

[148] Zhao, F. et al. Knockdown of a novel lincRNA AATBC suppresses proliferation and induces apoptosis in bladder cancer. *Oncotarget* **6**, 1064–1078 (2015). 25473900 10.18632/oncotarget.2833 PMC4359217

[149] Amodio, N. et al. Drugging the lncRNA MALAT1 via LNA gapmeR ASO inhibits gene expression of proteasome subunits and triggers anti-multiple myeloma activity. *Leukemia* **32**, 1948–1957 (2018). 29487387 10.1038/s41375-018-0067-3 PMC6127082

[150] Wu, X. C. et al. The NmrA-like family domain containing 1 pseudogene Loc344887 is amplified in gallbladder cancer and promotes epithelial-mesenchymal transition. *Chem. Biol. Drug Des.* **90**, 456–463 (2017). 28245089 10.1111/cbdd.12967

[151] Li, H. J. et al. Long non-coding RNA UCA1 promotes glutamine metabolism by targeting miR-16 in human bladder cancer. *Jpn J. Clin. Oncol.* **45**, 1055–1063 (2015). 26373319 10.1093/jjco/hyv132

[152] Ding, K., Liao, Y., Gong, D., Zhao, X. & Ji, W. Effect of long non-coding RNA H19 on oxidative stress and chemotherapy resistance of CD133+ cancer stem cells via the MAPK/ERK signaling pathway in hepatocellular carcinoma. *Biochem. Biophys. Res. Commun.* **502**, 194–201 (2018). 29800569 10.1016/j.bbrc.2018.05.143

[153] Chen, L. et al. LncRNA GAS5 regulates redox balance and dysregulates the cell cycle and apoptosis in malignant melanoma cells. *J. Cancer Res. Clin. Oncol.* **145**, 637–652 (2019). 30569211 10.1007/s00432-018-2820-4 PMC6394673

[154] Xu, J. et al. Paclitaxel promotes lung cancer cell apoptosis via MEG3-P53 pathway activation. *Biochem. Biophys. Res. Commun.* **504**, 123–128 (2018). 30173893 10.1016/j.bbrc.2018.08.142

[155] Zhang, H. Y., Zhang, B. W., Zhang, Z. B. & Deng, Q. J. Circular RNA TTBK2 regulates cell proliferation, invasion and ferroptosis via miR-761/ITGB8 axis in glioma. *Eur. Rev. Med. Pharmacol. Sci.* **24**, 2585–2600 (2020). 32196629 10.26355/eurrev_202003_20528

[156] Li, C., Tian, Y., Liang, Y. & Li, Q. Circ_0008035 contributes to cell proliferation and inhibits apoptosis and ferroptosis in gastric cancer via miR-599/EIF4A1 axis. *Cancer Cell Int.* **20**, 84 (2020). 32190008 10.1186/s12935-020-01168-0 PMC7076943

[157] Liu, Y. Y., Zhang, L. Y. & Du, W. Z. Circular RNA circ-PVT1 contributes to paclitaxel resistance of gastric cancer cells through the regulation of ZEB1 expression by sponging miR-124-3p. *Biosci. Rep.*10.1042/BSR20193045 (2019). 10.1042/BSR20193045 PMC6928529 31793989

[158] Zheng, S. R. et al. Human papillomavirus 16 E7 oncoprotein alters the expression profiles of circular RNAs in Caski cells. *J. Cancer* **9**, 3755–3764 (2018). 30405847 10.7150/jca.24253 PMC6216014

[159] Lorenz, C., Lunse, C. E. & Morl, M. tRNA modifications: impact on structure and thermal adaptation. *Biomolecules*10.3390/biom7020035 (2017). 10.3390/biom7020035 PMC5485724 28375166

[160] Hayano, M., Yang, W. S., Corn, C. K., Pagano, N. C. & Stockwell, B. R. Loss of cysteinyl-tRNA synthetase (CARS) induces the transsulfuration pathway and inhibits ferroptosis induced by cystine deprivation. *Cell Death Differ.* **23**, 270–278 (2016). 26184909 10.1038/cdd.2015.93 PMC4716307

[161] De Spirt, S. et al. Interplay between the chalcone cardamonin and selenium in the biosynthesis of Nrf2-regulated antioxidant enzymes in intestinal Caco-2 cells. *Free Radic. Biol. Med.* **91**, 164–171 (2016). 26698667 10.1016/j.freeradbiomed.2015.12.011

[162] Becker, N. P. et al. Hypoxia reduces and redirects selenoprotein biosynthesis. *Metallomics* **6**, 1079–1086 (2014). 24700164 10.1039/c4mt00004h

[163] Kipp, A. P., Frombach, J., Deubel, S. & Brigelius-Flohe, R. Selenoprotein W as biomarker for the efficacy of selenium compounds to act as source for selenoprotein biosynthesis. *Methods Enzymol.* **527**, 87–112 (2013). 23830627 10.1016/B978-0-12-405882-8.00005-2

[164] Hiramoto, K. et al. Myeloid lineage-specific deletion of antioxidant system enhances tumor metastasis. *Cancer Prev. Res.* **7**, 835–844 (2014). 10.1158/1940-6207.CAPR-14-0094 24866179

[165] Pathak, C., Jaiswal, Y. K. & Vinayak, M. Queuine promotes antioxidant defence system by activating cellular antioxidant enzyme activities in cancer. *Biosci. Rep.* **28**, 73–81 (2008). 18290765 10.1042/BSR20070011

[166] Tanaka, M., Han, S., Kupfer, P. A., Leumann, C. J. & Sonntag, W. E. An assay for RNA oxidation induced abasic sites using the aldehyde reactive probe. *Free Radic. Res.* **45**, 237–247 (2011). 21062214 10.3109/10715762.2010.535529 PMC3058411

[167] Li, W. et al. An internal ribosomal entry site mediates redox-sensitive translation of Nrf2. *Nucleic Acids Res.* **38**, 778–788 (2010). 19934254 10.1093/nar/gkp1048 PMC2817467

[168] Perez, M. J., Gonzalez-Sanchez, E., Gonzalez-Loyola, A., Gonzalez-Buitrago, J. M. & Marin, J. J. Mitochondrial genome depletion dysregulates bile acid- and paracetamol-induced expression of the transporters Mdr1, Mrp1 and Mrp4 in liver cells. *Br. J. Pharmacol.* **162**, 1686–1699 (2011). 21175587 10.1111/j.1476-5381.2010.01174.x PMC3081114

[169] Jayaraman, S. et al. The nuclear mitotic apparatus protein NuMA controls rDNA transcription and mediates the nucleolar stress response in a p53-independent manner. *Nucleic Acids Res.* **45**, 11725–11742 (2017). 28981686 10.1093/nar/gkx782 PMC5714241

[170] Yung, B. Y., Yang, Y. H. & Bor, A. M. Nucleolar protein B23 translocation after deferoxamine treatment in a human leukemia cell line. *Int. J. Cancer* **48**, 779–784 (1991). 2071236 10.1002/ijc.2910480524

[171] Halbach, R. et al. A satellite repeat-derived piRNA controls embryonic development of Aedes. *Nature* **580**, 274–277 (2020). 32269344 10.1038/s41586-020-2159-2 PMC7145458

[172] Liu, Y. et al. The emerging role of the piRNA/piwi complex in cancer. *Mol. Cancer* **18**, 123 (2019). 31399034 10.1186/s12943-019-1052-9 PMC6688334

[173] Zhang, L. et al. piR-31470 epigenetically suppresses the expression of glutathione S-transferase pi 1 in prostate cancer via DNA methylation. *Cell. Signal.* **67**, 109501 (2020). 31837464 10.1016/j.cellsig.2019.109501

[174] Oliveira, V. et al. The snoRNA target of t(4;14) in multiple myeloma regulates ribosome biogenesis. *FASEB Bioadv.* **1**, 404–414 (2019). 32095781 10.1096/fba.2018-00075 PMC6996358

[175] Wurth, L. et al. Hypermethylated-capped selenoprotein mRNAs in mammals. *Nucleic Acids Res.* **42**, 8663–8677 (2014). 25013170 10.1093/nar/gku580 PMC4117793

[176] Hider, R. C. & Kong, X. L. Glutathione: a key component of the cytoplasmic labile iron pool. *Biometals* **24**, 1179–1187 (2011). 21769609 10.1007/s10534-011-9476-8

[177] Watts, R. N. & Richardson, D. R. Nitrogen monoxide (no) and glucose: unexpected links between energy metabolism and no-mediated iron mobilization from cells. *J. Biol. Chem.* **276**, 4724–4732 (2001). 11078730 10.1074/jbc.M006318200

[178] Cole, S. P. & Deeley, R. G. Transport of glutathione and glutathione conjugates by MRP1. *Trends Pharmacol. Sci.* **27**, 438–446 (2006). 16820223 10.1016/j.tips.2006.06.008

[179] Watts, R. N., Hawkins, C., Ponka, P. & Richardson, D. R. Nitrogen monoxide (NO)-mediated iron release from cells is linked to NO-induced glutathione efflux via multidrug resistance-associated protein 1. *Proc. Natl Acad. Sci. USA* **103**, 7670–7675 (2006). 16679408 10.1073/pnas.0602515103 PMC1472503

[180] Agyeman, A. S. et al. Transcriptomic and proteomic profiling of KEAP1 disrupted and sulforaphane-treated human breast epithelial cells reveals common expression profiles. *Breast Cancer Res. Treat.* **132**, 175–187 (2012). 21597922 10.1007/s10549-011-1536-9 PMC3564494

[181] Harada, N. et al. Nrf2 regulates ferroportin 1-mediated iron efflux and counteracts lipopolysaccharide-induced ferroportin 1 mRNA suppression in macrophages. *Arch. Biochem. Biophys.* **508**, 101–109 (2011). 21303654 10.1016/j.abb.2011.02.001

[182] Yang, H. et al. Nrf1 and Nrf2 regulate rat glutamate-cysteine ligase catalytic subunit transcription indirectly via NF-kappaB and AP-1. *Mol. Cell. Biol.* **25**, 5933–5946 (2005). 15988009 10.1128/MCB.25.14.5933-5946.2005 PMC1168815

[183] Chan, J. Y. & Kwong, M. Impaired expression of glutathione synthetic enzyme genes in mice with targeted deletion of the Nrf2 basic-leucine zipper protein. *Biochim. et. Biophys. Acta* **1517**, 19–26 (2000). 10.1016/s0167-4781(00)00238-4 11118612

[184] Ishii, T. et al. Transcription factor Nrf2 coordinately regulates a group of oxidative stress-inducible genes in macrophages. *J. Biol. Chem.* **275**, 16023–16029 (2000). 10821856 10.1074/jbc.275.21.16023

[185] Ju, S. et al. The effect and mechanism of miR-210 in down-regulating the autophagy of lung cancer cells. *Pathol. Res. Pract.* **215**, 453–458 (2019). 30573163 10.1016/j.prp.2018.12.018

[186] Wang, Z., Deng, M., Liu, Z. & Wu, S. Hypoxia-induced miR-210 promoter demethylation enhances proliferation, autophagy and angiogenesis of schwannoma cells. *Oncol. Rep.* **37**, 3010–3018 (2017). 28440459 10.3892/or.2017.5511

[187] Wang, J. et al. The long noncoding RNA H19 promotes tamoxifen resistance in breast cancer via autophagy. *J. Hematol. Oncol.* **12**, 81 (2019). 31340867 10.1186/s13045-019-0747-0 PMC6657081

[188] Wang, M. et al. Long non-coding RNA H19 confers 5-Fu resistance in colorectal cancer by promoting SIRT1-mediated autophagy. *Cell Death Dis.* **9**, 1149 (2018). 30451820 10.1038/s41419-018-1187-4 PMC6242979

[189] Saenen, N. D. et al. Air pollution-induced placental alterations: an interplay of oxidative stress, epigenetics, and the aging phenotype? *Clin. Epigenet.* **11**, 124 (2019). 10.1186/s13148-019-0688-z PMC6749657 31530287

[190] Rippo, M. R. et al. MitomiRs in human inflamm-aging: a hypothesis involving miR-181a, miR-34a and miR-146a. *Exp. Gerontol.* **56**, 154–163 (2014). 24607549 10.1016/j.exger.2014.03.002

[191] Su, P. et al. The role of autophagy in modulation of neuroinflammation in microglia. *Neuroscience* **319**, 155–167 (2016). 26827945 10.1016/j.neuroscience.2016.01.035

[192] Li, P. A., Hou, X. & Hao, S. Mitochondrial biogenesis in neurodegeneration. *J. Neurosci. Res.* **95**, 2025–2029 (2017). 28301064 10.1002/jnr.24042

[193] Guo, L. et al. eIF2alpha promotes vascular remodeling via autophagy in monocrotaline-induced pulmonary arterial hypertension rats. *Drug Des. Dev. Ther.* **13**, 2799–2809 (2019). 10.2147/DDDT.S213817 PMC6698179 31496656

[194] Lu, C., Yang, Y., Zhu, Y., Lv, S. & Zhang, J. An intervention target for myocardial fibrosis: autophagy. *BioMed. Res. Int.* **2018**, 6215916 (2018). 29850542 10.1155/2018/6215916 PMC5911341

[195] Rojo de la Vega, M., Chapman, E. & Zhang, D. D. NRF2 and the hallmarks of cancer. *Cancer Cell* **34**, 21–43 (2018). 29731393 10.1016/j.ccell.2018.03.022 PMC6039250

[196] Dodson, M., Castro-Portuguez, R. & Zhang, D. D. NRF2 plays a critical role in mitigating lipid peroxidation and ferroptosis. *Redox Biol.* **23**, 101107 (2019). 30692038 10.1016/j.redox.2019.101107 PMC6859567

[197] Sarmento-Ribeiro, A. B., Scorilas, A., Goncalves, A. C., Efferth, T. & Trougakos, I. P. The emergence of drug resistance to targeted cancer therapies: clinical evidence. *Drug Resist. Updates* **47**, 100646 (2019). 10.1016/j.drup.2019.100646 31733611

[198] Park, E. & Chung, S. W. ROS-mediated autophagy increases intracellular iron levels and ferroptosis by ferritin and transferrin receptor regulation. *Cell Death Dis.* **10**, 822 (2019). 31659150 10.1038/s41419-019-2064-5 PMC6817894

[199] Banerjee, N. et al. Plum polyphenols inhibit colorectal aberrant crypt foci formation in rats: potential role of the miR-143/protein kinase B/mammalian target of rapamycin axis. *Nutr. Res.* **36**, 1105–1113 (2016). 27865352 10.1016/j.nutres.2016.06.008

[200] Zhang, X. et al. miR-513a-3p sensitizes human lung adenocarcinoma cells to chemotherapy by targeting GSTP1. *Lung Cancer* **77**, 488–494 (2012). 22749944 10.1016/j.lungcan.2012.05.107

[201] Li, A., Yang, C. & Hu, M. Viability of colon tumor cells in insufficient-nutritional condition is reduced by MiR-133b through regulating expression of GSTP1. *J. Sichuan Univ.* **48**, 699–704 (2017). 29130660

[202] Zong, C., Wang, J. & Shi, T. M. MicroRNA 130b enhances drug resistance in human ovarian cancer cells. *Tumour Biol.* **35**, 12151–12156 (2014). 25155039 10.1007/s13277-014-2520-x

[203] Sun, K. X., Jiao, J. W., Chen, S., Liu, B. L. & Zhao, Y. MicroRNA-186 induces sensitivity of ovarian cancer cells to paclitaxel and cisplatin by targeting ABCB1. *J. Ovarian Res.* **8**, 80 (2015). 26626440 10.1186/s13048-015-0207-6 PMC4667519

[204] Benassi, B., Marani, M., Loda, M. & Blandino, G. USP2a alters chemotherapeutic response by modulating redox. *Cell Death Dis.* **4**, e812 (2013). 24071644 10.1038/cddis.2013.289 PMC3789164

[205] Qin, Z. et al. Upregulation of xCT by KSHV-encoded microRNAs facilitates KSHV dissemination and persistence in an environment of oxidative stress. *PLoS Pathog.* **6**, e1000742 (2010). 20126446 10.1371/journal.ppat.1000742 PMC2813276

[206] Tili, E. et al. The down-regulation of miR-125b in chronic lymphocytic leukemias leads to metabolic adaptation of cells to a transformed state. *Blood* **120**, 2631–2638 (2012). 22723551 10.1182/blood-2012-03-415737 PMC3460685

[207] Sun, A. G., Meng, F. G. & Wang, M. G. CISD2 promotes the proliferation of glioma cells via suppressing beclin1mediated autophagy and is targeted by microRNA449a. *Mol. Med. Rep.* **16**, 7939–7948 (2017). 28983596 10.3892/mmr.2017.7642 PMC5779876

[208] Zhang, L. et al. MicroRNA-related genetic variants in iron regulatory genes, dietary iron intake, microRNAs and lung cancer risk. *Ann. Oncol.* **28**, 1124–1129 (2017). 28453699 10.1093/annonc/mdx046 PMC5834125

[209] Saenz-de-Santa-Maria, I. et al. Clinically relevant HIF-1alpha-dependent metabolic reprogramming in oropharyngeal squamous cell carcinomas includes coordinated activation of CAIX and the miR-210/ISCU signaling axis, but not MCT1 and MCT4 upregulation. *Oncotarget* **8**, 13730–13746 (2017). 28099149 10.18632/oncotarget.14629 PMC5355133

[210] McCormick, R. I. et al. miR-210 is a target of hypoxia-inducible factors 1 and 2 in renal cancer, regulates ISCU and correlates with good prognosis. *Br. J. Cancer* **108**, 1133–1142 (2013). 23449350 10.1038/bjc.2013.56 PMC3619073

[211] Neal, C. S., Michael, M. Z., Rawlings, L. H., Van der Hoek, M. B. & Gleadle, J. M. The VHL-dependent regulation of microRNAs in renal cancer. *BMC Med.* **8**, 64 (2010). 20964835 10.1186/1741-7015-8-64 PMC2978113

[212] Chew, S. H. & Toyokuni, S. Malignant mesothelioma as an oxidative stress-induced cancer: an update. *Free Radic. Biol. Med.* **86**, 166–178 (2015). 25975982 10.1016/j.freeradbiomed.2015.05.002

[213] Thakral, S. & Ghoshal, K. miR-122 is a unique molecule with great potential in diagnosis, prognosis of liver disease, and therapy both as miRNA mimic and antimir. *Curr. Gene Ther.* **15**, 142–150 (2015). 25537773 10.2174/1566523214666141224095610 PMC4439190

[214] Ren, J. et al. LF-MF inhibits iron metabolism and suppresses lung cancer through activation of P53-miR-34a-E2F1/E2F3 pathway. *Sci. Rep.* **7**, 749 (2017). 28389657 10.1038/s41598-017-00913-2 PMC5429732

[215] Petrelli, A. et al. MicroRNA/gene profiling unveils early molecular changes and nuclear factor erythroid related factor 2 (NRF2) activation in a rat model recapitulating human hepatocellular carcinoma (HCC). *Hepatology* **59**, 228–241 (2014). 23857252 10.1002/hep.26616

[216] Eades, G., Yang, M., Yao, Y., Zhang, Y. & Zhou, Q. miR-200a regulates Nrf2 activation by targeting Keap1 mRNA in breast cancer cells. *J. Biol. Chem.* **286**, 40725–40733 (2011). 21926171 10.1074/jbc.M111.275495 PMC3220489

[217] Gu, S., Lai, Y., Chen, H., Liu, Y. & Zhang, Z. miR-155 mediates arsenic trioxide resistance by activating Nrf2 and suppressing apoptosis in lung cancer cells. *Sci. Rep.* **7**, 12155 (2017). 28939896 10.1038/s41598-017-06061-x PMC5610328

[218] Gao, A. M., Zhang, X. Y. & Ke, Z. P. Apigenin sensitizes BEL-7402/ADM cells to doxorubicin through inhibiting miR-101/Nrf2 pathway. *Oncotarget* **8**, 82085–82091 (2017). 29137246 10.18632/oncotarget.18294 PMC5669872

[219] Qu, J., Zhang, L., Li, L. & Su, Y. miR-148b functions as a tumor suppressor by targeting endoplasmic reticulum metallo protease 1 in human endometrial cancer cells. *Oncol. Res.* **27**, 81–88 (2018). 29523216 10.3727/096504018X15202988139874 PMC7848254

[220] Chen, G. et al. Lico A causes ER stress and apoptosis via up-regulating miR-144-3p in human lung cancer cell line H292. *Front. Pharmacol.* **9**, 837 (2018). 30108506 10.3389/fphar.2018.00837 PMC6079201

[221] Sun, X., Liu, D., Xue, Y. & Hu, X. Enforced miR-144-3p expression as a non-invasive biomarker for the acute myeloid leukemia patients mainly by targeting NRF2. *Clin. Lab.* **63**, 679–687 (2017). 28397476 10.7754/Clin.Lab.2016.161116

[222] Cortez, M. A. et al. Therapeutic delivery of miR-200c enhances radiosensitivity in lung cancer. *Mol. Ther.* **22**, 1494–1503 (2014). 24791940 10.1038/mt.2014.79 PMC4435581

[223] Singh, B., Ronghe, A. M., Chatterjee, A., Bhat, N. K. & Bhat, H. K. MicroRNA-93 regulates NRF2 expression and is associated with breast carcinogenesis. *Carcinogenesis* **34**, 1165–1172 (2013). 23492819 10.1093/carcin/bgt026 PMC3643421

[224] Papp, D. et al. The NRF2-related interactome and regulome contain multifunctional proteins and fine-tuned autoregulatory loops. *FEBS Lett.* **586**, 1795–1802 (2012). 22641035 10.1016/j.febslet.2012.05.016 PMC7511993

[225] Do, M. T., Kim, H. G., Choi, J. H. & Jeong, H. G. Metformin induces microRNA-34a to downregulate the Sirt1/Pgc-1alpha/Nrf2 pathway, leading to increased susceptibility of wild-type p53 cancer cells to oxidative stress and therapeutic agents. *Free Radic. Biol. Med.* **74**, 21–34 (2014). 24970682 10.1016/j.freeradbiomed.2014.06.010

[226] Trivedi, M. et al. MicroRNA-34a encapsulated in hyaluronic acid nanoparticles induces epigenetic changes with altered mitochondrial bioenergetics and apoptosis in non-small-cell lung cancer cells. *Sci. Rep.* **7**, 3636 (2017). 28623259 10.1038/s41598-017-02816-8 PMC5473901

[227] Li, C. et al. Deregulation of UCA1 expression may be involved in the development of chemoresistance to cisplatin in the treatment of non-small-cell lung cancer via regulating the signaling pathway of microRNA-495/NRF2. *J. Cell. Physiol.*10.1002/jcp.29266 (2019). 31583720 10.1002/jcp.29266 PMC13482702

[228] Joo, M. S., Lee, C. G., Koo, J. H. & Kim, S. G. miR-125b transcriptionally increased by Nrf2 inhibits AhR repressor, which protects kidney from cisplatin-induced injury. *Cell death Dis.* **4**, e899 (2013). 24176857 10.1038/cddis.2013.427 PMC3920955

[229] Chen, P. et al. Curcumin promotes osteosarcoma cell death by activating miR-125a/ERRalpha signal pathway. *J. Cell. Biochem.* **118**, 74–81 (2017). 27231954 10.1002/jcb.25612

[230] Sosa, V. et al. Oxidative stress and cancer: an overview. *Ageing Res. Rev.* **12**, 376–390 (2013). 23123177 10.1016/j.arr.2012.10.004

[231] Pajic, M. et al. miR-139-5p modulates radiotherapy resistance in breast cancer by repressing multiple gene networks of DNA repair and ROS defense. *Cancer Res.* **78**, 501–515 (2018). 29180477 10.1158/0008-5472.CAN-16-3105

[232] Zhang, H. M. et al. miR-146b-5p within BCR-ABL1-positive microvesicles promotes leukemic transformation of hematopoietic cells. *Cancer Res.* **76**, 2901–2911 (2016). 27013199 10.1158/0008-5472.CAN-15-2120

[233] Xue, G. et al. c-Myc-mediated repression of miR-15-16 in hypoxia is induced by increased HIF-2alpha and promotes tumor angiogenesis and metastasis by upregulating FGF2. *Oncogene* **34**, 1393–1406 (2015). 24704828 10.1038/onc.2014.82

[234] Liu, Q. et al. miR-155 regulates glioma cells invasion and chemosensitivity by p38 isforms in vitro. *J. Cell. Biochem.* **116**, 1213–1221 (2015). 25535908 10.1002/jcb.25073

[235] Wang, P. et al. Micro-RNA-155 is induced by K-Ras oncogenic signal and promotes ROS stress in pancreatic cancer. *Oncotarget* **6**, 21148–21158 (2015). 26020803 10.18632/oncotarget.4125 PMC4673256

[236] Wu, L. et al. Polygonatum odoratum lectin induces apoptosis and autophagy by regulation of microRNA-1290 and microRNA-15a-3p in human lung adenocarcinoma A549 cells. *Int. J. Biol. Macromol.* **85**, 217–226 (2016). 26562549 10.1016/j.ijbiomac.2015.11.014

[237] Kim, S. Y., Lee, Y. H. & Bae, Y. S. MiR-186, miR-216b, miR-337-3p, and miR-760 cooperatively induce cellular senescence by targeting alpha subunit of protein kinase CKII in human colorectal cancer cells. *Biochem. Biophys. Res. Commun.* **429**, 173–179 (2012). 23137536 10.1016/j.bbrc.2012.10.117

[238] Kwon, J. E., Kim, B. Y., Kwak, S. Y., Bae, I. H. & Han, Y. H. Ionizing radiation-inducible microRNA miR-193a-3p induces apoptosis by directly targeting Mcl-1. *Apoptosis* **18**, 896–909 (2013). 23546867 10.1007/s10495-013-0841-7

[239] Tang, T. et al. Up-regulation of miR-210 induced by a hypoxic microenvironment promotes breast cancer stem cells metastasis, proliferation, and self-renewal by targeting E-cadherin. *FASEB J.*10.1096/fj.201801013R (2018). 10.1096/fj.201801013R 30188754

[240] Nozoe, T., Honda, M., Inutsuka, S., Yasuda, M. & Korenaga, D. Significance of immunohistochemical expression of manganese superoxide dismutase as a marker of malignant potential in colorectal carcinoma. *Oncol. Rep.* **10**, 39–43 (2003). 12469142

[241] Pant, K. et al. Butyrate induces ROS-mediated apoptosis by modulating miR-22/SIRT-1 pathway in hepatic cancer cells. *Redox Biol.* **12**, 340–349 (2017). 28288414 10.1016/j.redox.2017.03.006 PMC5350572

[242] Sun, X., Li, Y., Zheng, M., Zuo, W. & Zheng, W. MicroRNA-223 increases the sensitivity of triple-negative breast cancer stem cells to TRAIL-induced apoptosis by targeting HAX-1. *PLoS ONE* **11**, e0162754 (2016). 27618431 10.1371/journal.pone.0162754 PMC5019415

[243] Jiang, W. et al. MicroRNA-26a-5p and microRNA-23b-3p up-regulate peroxiredoxin III in acute myeloid leukemia. *Leuk. Lymphoma* **56**, 460–471 (2015). 24828865 10.3109/10428194.2014.924115 PMC4364273

[244] Jung, J. H. et al. NEDD9 inhibition by miR-25-5p activation is critically involved in co-treatment of melatonin- and pterostilbene-induced apoptosis in colorectal cancer cells. *Cancers*10.3390/cancers11111684 (2019). 10.3390/cancers11111684 PMC6895813 31671847

[245] Guo, X. et al. Immunosuppressive effects of hypoxia-induced glioma exosomes through myeloid-derived suppressor cells via the miR-10a/Rora and miR-21/Pten pathways. *Oncogene* **37**, 4239–4259 (2018). 29713056 10.1038/s41388-018-0261-9

[246] Wang, J., Jiao, Y., Cui, L. & Jiang, L. miR-30 functions as an oncomiR in gastric cancer cells through regulation of P53-mediated mitochondrial apoptotic pathway. *Biosci. Biotechnol. Biochem.* **81**, 119–126 (2017). 27729002 10.1080/09168451.2016.1238294

[247] Tu, L. et al. MiR-34c acts as a tumor suppressor in non-small cell lung cancer by inducing endoplasmic reticulum stress through targeting HMGB1. *OncoTargets Ther.* **12**, 5729–5739 (2019). 10.2147/OTT.S206932 PMC6647009 31410019

[248] Sahu, N. et al. Functional screening implicates miR-371-3p and peroxiredoxin 6 in reversible tolerance to cancer drugs. *Nat. Commun.* **7**, 12351 (2016). 27484502 10.1038/ncomms12351 PMC4976141

[249] He, Z. et al. MiR-422a regulates cellular metabolism and malignancy by targeting pyruvate dehydrogenase kinase 2 in gastric cancer. *Cell Death Dis.* **9**, 505 (2018). 29725130 10.1038/s41419-018-0564-3 PMC5938701

[250] Huang, H. L. et al. MiR-4673 modulates paclitaxel-induced oxidative stress and loss of mitochondrial membrane potential by targeting 8-oxoguanine-DNA glycosylase-1. *Cell. Physiol. Biochem.* **42**, 889–900 (2017). 28647734 10.1159/000478644

[251] Bublik, D. R. et al. Regulatory module involving FGF13, miR-504, and p53 regulates ribosomal biogenesis and supports cancer cell survival. *Proc. Natl Acad. Sci. USA* **114**, E496–E505 (2017). 27994142 10.1073/pnas.1614876114 PMC5278483

[252] Yin, M. et al. Selective killing of lung cancer cells by miRNA-506 molecule through inhibiting NF-kappaB p65 to evoke reactive oxygen species generation and p53 activation. *Oncogene* **34**, 691–703 (2015). 24469051 10.1038/onc.2013.597

[253] Song, Y. H. et al. MicroRNA-509-5p functions as an anti-oncogene in breast cancer via targeting SOD2. *Eur. Rev. Med. Pharmacol. Sci.* **21**, 3617–3625 (2017). 28925482

[254] Xu, X. et al. A signaling pathway consisting of miR-551b, catalase and MUC1 contributes to acquired apoptosis resistance and chemoresistance. *Carcinogenesis* **35**, 2457–2466 (2014). 25085901 10.1093/carcin/bgu159 PMC4216053

[255] Gomez de Cedron, M. et al. MicroRNA-661 modulates redox and metabolic homeostasis in colon cancer. *Mol. Oncol.* **11**, 1768–1787 (2017). 28981199 10.1002/1878-0261.12142 PMC5709620

[256] Cardin, R. et al. Oxidative DNA damage correlates with cell immortalization and mir-92 expression in hepatocellular carcinoma. *BMC Cancer* **12**, 177 (2012). 22587342 10.1186/1471-2407-12-177 PMC3420318

[257] Chen, P. H. et al. The inhibition of microRNA-128 on IGF-1-activating mTOR signaling involves in temozolomide-induced glioma cell apoptotic death. *PLoS ONE* **11**, e0167096 (2016). 27893811 10.1371/journal.pone.0167096 PMC5125683

[258] Li, Q. et al. Insulin regulates glucose consumption and lactate production through reactive oxygen species and pyruvate kinase M2. *Oxid. Med. Cell. Longev.* **2014**, 504953 (2014). 24895527 10.1155/2014/504953 PMC4034658

[259] Cha, J. A. et al. miR-211 plays a critical role in Cnidium officinale Makino extract-induced, ROS/ER stress-mediated apoptosis in U937 and U266 cells. *Int. J. Mol. Sci.*10.3390/ijms19030865 (2018). 10.3390/ijms19030865 PMC5877726 29543750

[260] Chen, Y. F. et al. MicroRNA-211 enhances the oncogenicity of carcinogen-induced oral carcinoma by repressing TCF12 and increasing antioxidant activity. *Cancer Res.* **76**, 4872–4886 (2016). 27221705 10.1158/0008-5472.CAN-15-1664

[261] Bao, B. et al. Targeting CSCs in tumor microenvironment: the potential role of ROS-associated miRNAs in tumor aggressiveness. *Curr. Stem Cell Res. Ther.* **9**, 22–35 (2014). 23957937 10.2174/1574888x113089990053 PMC4493722

[262] Li, B. et al. miR-221/222 promote cancer stem-like cell properties and tumor growth of breast cancer via targeting PTEN and sustained Akt/NF-kappaB/COX-2 activation. *Chem. Biol. Interact.* **277**, 33–42 (2017). 28844858 10.1016/j.cbi.2017.08.014

[263] Fulciniti, M. et al. miR-23b/SP1/c-myc forms a feed-forward loop supporting multiple myeloma cell growth. *Blood Cancer J.* **6**, e380 (2016). 26771806 10.1038/bcj.2015.106 PMC4742623

[264] Liu, W. et al. miR-23b targets proline oxidase, a novel tumor suppressor protein in renal cancer. *Oncogene* **29**, 4914–4924 (2010). 20562915 10.1038/onc.2010.237 PMC4398970

[265] Kurinna, S. et al. A novel Nrf2-miR-29-desmocollin-2 axis regulates desmosome function in keratinocytes. *Nat. Commun.* **5**, 5099 (2014). 25283360 10.1038/ncomms6099

[266] Hou, M., Zuo, X., Li, C., Zhang, Y. & Teng, Y. Mir-29b regulates oxidative stress by targeting SIRT1 in ovarian cancer cells. *Cell. Physiol. Biochem.* **43**, 1767–1776 (2017). 29050034 10.1159/000484063

[267] Kim, S. M., Hur, D. Y., Hong, S. W. & Kim, J. H. EBV-encoded EBNA1 regulates cell viability by modulating miR34a-NOX2-ROS signaling in gastric cancer cells. *Biochem. Biophys. Res. Commun.* **494**, 550–555 (2017). 29061308 10.1016/j.bbrc.2017.10.095

[268] Hou, W., Tian, Q., Steuerwald, N. M., Schrum, L. W. & Bonkovsky, H. L. The let-7 microRNA enhances heme oxygenase-1 by suppressing Bach1 and attenuates oxidant injury in human hepatocytes. *Biochim. et. Biophys. Acta* **1819**, 1113–1122 (2012). 10.1016/j.bbagrm.2012.06.001 PMC3480558 22698995

[269] Chang, M. et al. Suppression of SIRT6 by miR-33a facilitates tumor growth of glioma through apoptosis and oxidative stress resistance. *Oncol. Rep.* **38**, 1251–1258 (2017). 28677777 10.3892/or.2017.5780

[270] Pradhan, A. K. et al. MDA-7/IL-24 regulates the miRNA processing enzyme DICER through downregulation of MITF. *Proc. Natl Acad. Sci. USA* **116**, 5687–5692 (2019). 30842276 10.1073/pnas.1819869116 PMC6431152

[271] Zhang, H. et al. Electrochemiluminescence-microscopy for microRNA imaging in single cancer cell combined with chemotherapy-photothermal therapy. *Anal. Chem.* **91**, 12581–12586 (2019). 31539224 10.1021/acs.analchem.9b03694

[272] Strickertsson, J. A., Rasmussen, L. J. & Friis-Hansen, L. Enterococcus faecalis infection and reactive oxygen species down-regulates the miR-17-92 cluster in gastric adenocarcinoma cell culture. *Genes* **5**, 726–738 (2014). 25170597 10.3390/genes5030726 PMC4198927

[273] Ebi, H. et al. Counterbalance between RB inactivation and miR-17-92 overexpression in reactive oxygen species and DNA damage induction in lung cancers. *Oncogene* **28**, 3371–3379 (2009). 19597473 10.1038/onc.2009.201

[274] Luo, J., Chen, P., Xie, W. & Wu, F. MicroRNA-138 inhibits cell proliferation in hepatocellular carcinoma by targeting Sirt1. *Oncol. Rep.* **38**, 1067–1074 (2017). 28677784 10.3892/or.2017.5782

[275] Magenta, A. et al. miR-200c is upregulated by oxidative stress and induces endothelial cell apoptosis and senescence via ZEB1 inhibition. *Cell Death Differ.* **18**, 1628–1639 (2011). 21527937 10.1038/cdd.2011.42 PMC3172120

[276] Xiao, Y. et al. p38/p53/miR-200a-3p feedback loop promotes oxidative stress-mediated liver cell death. *Cell Cycle* **14**, 1548–1558 (2015). 25789565 10.1080/15384101.2015.1026491 PMC4615042

[277] Huang, C. et al. The effects of ultrasound exposure on P-glycoprotein-mediated multidrug resistance in vitro and in vivo. *J. Exp. Clin. Cancer Res.* **37**, 232 (2018). 30231924 10.1186/s13046-018-0900-6 PMC6149229

[278] Baker, J. R. et al. Oxidative stress dependent microRNA-34a activation via PI3Kalpha reduces the expression of sirtuin-1 and sirtuin-6 in epithelial cells. *Sci. Rep.* **6**, 35871 (2016). 27767101 10.1038/srep35871 PMC5073335

[279] Chakraborty, S. et al. Restoration of p53/miR-34a regulatory axis decreases survival advantage and ensures Bax-dependent apoptosis of non-small cell lung carcinoma cells. *FEBS Lett.* **588**, 549–559 (2014). 24444609 10.1016/j.febslet.2013.11.040

[280] Liu, L. et al. MicroRNA-20a-mediated loss of autophagy contributes to breast tumorigenesis by promoting genomic damage and instability. *Oncogene* **36**, 5874–5884 (2017). 28628113 10.1038/onc.2017.193 PMC5658668

[281] Li, W. et al. Astragalin reduces hexokinase 2 through increasing miR-125b to inhibit the proliferation of hepatocellular carcinoma cells in vitro and in vivo. *J. Agric. Food Chem.* **65**, 5961–5972 (2017). 28654261 10.1021/acs.jafc.7b02120

[282] Shukla, K. et al. MicroRNA-30c-2-3p negatively regulates NF-kappaB signaling and cell cycle progression through downregulation of TRADD and CCNE1 in breast cancer. *Mol. Oncol.* **9**, 1106–1119 (2015). 25732226 10.1016/j.molonc.2015.01.008 PMC5528752

[283] Guo, J. et al. miR-346 functions as a pro-survival factor under ER stress by activating mitophagy. *Cancer Lett.* **413**, 69–81 (2018). 29107113 10.1016/j.canlet.2017.10.030

[284] Vera-Puente, O. et al. MAFG is a potential therapeutic target to restore chemosensitivity in cisplatin-resistant cancer cells by increasing reactive oxygen species. *Transl. Res.* **200**, 1–17 (2018). 30053382 10.1016/j.trsl.2018.06.005 PMC7787305

[285] Zou, S., Rao, Y. & Chen, W. miR-885-5p plays an accomplice role in liver cancer by instigating TIGAR expression via targeting its promoter. *Biotechnol. Appl. Biochem.* **66**, 763–771 (2019). 31119791 10.1002/bab.1767

[286] Noratto, G. D., Jutooru, I., Safe, S., Angel-Morales, G. & Mertens-Talcott, S. U. The drug resistance suppression induced by curcuminoids in colon cancer SW-480 cells is mediated by reactive oxygen species-induced disruption of the microRNA-27a-ZBTB10-Sp axis. *Mol. Nutr. Food Res.* **57**, 1638–1648 (2013). 23471840 10.1002/mnfr.201200609

[287] Ishimoto, T. et al. Macrophage-derived reactive oxygen species suppress miR-328 targeting CD44 in cancer cells and promote redox adaptation. *Carcinogenesis* **35**, 1003–1011 (2014). 24318997 10.1093/carcin/bgt402

[288] Kang, H. et al. Downregulation of microRNA-362-3p and microRNA-329 promotes tumor progression in human breast cancer. *Cell Death Differ.* **23**, 484–495 (2016). 26337669 10.1038/cdd.2015.116 PMC5072442

[289] Bountali, A., Tonge, D. P. & Mourtada-Maarabouni, M. RNA sequencing reveals a key role for the long non-coding RNA MIAT in regulating neuroblastoma and glioblastoma cell fate. *Int. J. Biol. macromol.* **130**, 878–891 (2019). 30836187 10.1016/j.ijbiomac.2019.03.005

[290] Tobe, R. et al. High error rates in selenocysteine insertion in mammalian cells treated with the antibiotic doxycycline, chloramphenicol, or geneticin. *J. Biol. Chem.* **288**, 14709–14715 (2013). 23589299 10.1074/jbc.M112.446666 PMC3663496

[291] Schonberg, S. A. et al. Evidence that changes in Se-glutathione peroxidase levels affect the sensitivity of human tumour cell lines to n-3 fatty acids. *Carcinogenesis* **18**, 1897–1904 (1997). 9363997 10.1093/carcin/18.10.1897

[292] Shimada, K. et al. A novel human AlkB homologue, ALKBH8, contributes to human bladder cancer progression. *Cancer Res.* **69**, 3157–3164 (2009). 19293182 10.1158/0008-5472.CAN-08-3530

[293] Zolla, L. & Timperio, A. M. Involvement of active oxygen species in protein and oligonucleotide degradation induced by nitrofurans. *Biochem. Cell Biol.* **83**, 166–175 (2005). 15864325 10.1139/o05-023

[294] Gunderson, S. I., Vagner, S., Polycarpou-Schwarz, M. & Mattaj, I. W. Involvement of the carboxyl terminus of vertebrate poly(A) polymerase in U1A autoregulation and in the coupling of splicing and polyadenylation. *Genes Dev.* **11**, 761–773 (1997). 9087430 10.1101/gad.11.6.761

[295] Wan, L., Ottinger, E., Cho, S. & Dreyfuss, G. Inactivation of the SMN complex by oxidative stress. *Mol. Cell* **31**, 244–254 (2008). 18657506 10.1016/j.molcel.2008.06.004 PMC2867055

