# Supplementary material for: Crosstalk between noncoding RNAs and ferroptosis: new dawn for overcoming cancer progression
Source: Cell Death Dis. 2020 Jul 24;11(7):580. doi: 10.1038/s41419-020-02772-8 (PMC7381619; doi:10.1038/s41419-020-02772-8)
Supplement: Supplementary file 1 — Supplementary Table 1 [file 41419_2020_2772_MOESM1_ESM.docx]

**Supplementary Table 1. Summary of ferroptosis associated miRNAs and lncRNAs in cancer**

| Type of RNA | Name | Associated cancer types | Targets | Influence to ferroptosis | References |
| --- | --- | --- | --- | --- | --- |
| MiRNA | *miR-137* | Melanoma | SLC1A5 | Up | [^31^](#_ENREF_31) |
|  | *miR-6852* | Lung cancer | CBS | Up | [^32^](#_ENREF_32) |
|  | *miR-7-5p* | Ovarian cancer, colorectal cancer | Mitoferrin | Up | [^30^](#_ENREF_30) |
|  | *miR-9* | Melanoma | Glutamic-oxaloacetic transaminase | Unknown | [^31^](#_ENREF_31) |
| LncRNA | *P53rra* | Breast cancer | P53 | Up | [^140^](#_ENREF_140) |
|  | *P53rra* | Lung cancer | P53 | Up | [^140^](#_ENREF_140) |
|  | *Gabpb1-as1* | Hepatocellular carcinoma | Peroxiredoxin-5 | Up | [^141^](#_ENREF_141) |
|  | *Linc00336* | Lung cancer | CBS | Down | [^32^](#_ENREF_32) |
