# Supplementary material for: Crosstalk between noncoding RNAs and ferroptosis: new dawn for overcoming cancer progression
Source: Cell Death Dis. 2020 Jul 24;11(7):580. doi: 10.1038/s41419-020-02772-8 (PMC7381619; doi:10.1038/s41419-020-02772-8)
Supplement: Supplementary file 2 — Supplementary Table 2 [file 41419_2020_2772_MOESM2_ESM.docx]

**Supplementary Table 2. Summary of ferroptosis associated circRNA, snRNAs, snoRNAs, tRNAs, rRNAs and piRNAs in cancer**

| Type of RNA | Control point | Name | Associated cancer type | Target | Influence to control point | Model of evidence | Reference |
| --- | --- | --- | --- | --- | --- | --- | --- |
| CircularRNA | Ferroptosis | *CircTTBK2* | Glioma | *miR-761*-ITGB8 axis | Down | Cell culture, animal models | [^155^](#_ENREF_155) |
|  |  | *Circ0008035* | Gastric cancer | *miR-599*-EIF4A1 axis | Down | Cell culture, animal models | [^156^](#_ENREF_156) |
|  | GSH | *CircPVT1* | Gastric cancer | GST-π | Down | Cell culture, animal models | [^157^](#_ENREF_157) |
|  | Iron | *Circ0008035* | Gastric cancer | *miR-599*-EIF4A1 axis | Down | Cell culture, animal models | [^156^](#_ENREF_156) |
| TRNA | Ferroptosis | *Cysteinyl-tRNA* | Fibrosarcoma, rhabdomyosarcoma, pancreatic carcinoma | CARS | Up | Cell culture | [^160^](#_ENREF_160) |
|  | GSH | *Selenocysteine-tRNA* | Hepatocellular carcinoma, colorectal cancer | GPX1 | Down | Cell culture | [^163^](#_ENREF_163) |
|  |  | *Selenocysteine-tRNA* | Hepatocellular carcinoma | GPX4 | Down | Cell culture | [^162^](#_ENREF_162) |
|  |  | *Selenocysteine-tRNA* | Breast cancer | GPX1 | Down | Cell culture, animal models | [^290^](#_ENREF_290) |
|  |  | *Selenocysteine-tRNA* | Lymphoma | GPX, GR | Down | Cell culture, animal models | [^165^](#_ENREF_165) |
|  |  | *Selenocysteine-tRNA* | Lung cancer | GPX | Down | Cell culture | [^291^](#_ENREF_291) |
|  | ROS | *tRNA* | Urothelial carcinoma | NOX-1 | Up | Cell culture, animal models | [^292^](#_ENREF_292) |
|  |  | *Selenocysteine-tRNA* | Lung cancer | Selenoenzymes | Down | Cell culture, animal models | [^164^](#_ENREF_164) |
|  |  | *Queuine-modified tRNA* | Lymphoma | GPX, GR | Down | Cell culture, animal models | [^165^](#_ENREF_165) |
| RRNA | Iron | *rRNA* | Leukemia | B23 | Induced by iron | Cell culture | [^170^](#_ENREF_170) |
|  | NRF2 | 18S*rRNA* | Hepatocellular carcinoma | NRF2 | Up | Cell culture, animal models | [^168^](#_ENREF_168) |
|  |  | 18S*rRNA* | Cervical cancer | NRF2 | Up | Cell culture | [^167^](#_ENREF_167) |
|  | ROS | 18S*rRNA* | Hepatocellular carcinoma | NRF2 | Up | Cell culture, animal models | [^168^](#_ENREF_168) |
|  |  | 18S and 28S *rRNA* | Breast cancer | NUMA | Down | Cell culture | [^169^](#_ENREF_169) |
|  |  | *rRNA* | Colorectal cancer | - | Inhibited by ROS | Cell culture, animal models | [^293^](#_ENREF_293) |
| SnoRNA | GSH | *U3* | Cervical cancer, osteosarcoma | TGS1 | Down | Cell culture | [^175^](#_ENREF_175) |
|  | ROS | *Aca11* | Multiple myeloma | Unknown | Up | Cell culture | [^174^](#_ENREF_174) |
| SnRNA | GSH | *U1* | Cervical cancer | GST | Up | Cell culture | [^294^](#_ENREF_294) |
|  |  | *U2* | Cervical cancer, osteosarcoma | TGS1 | Down | Cell culture | [^175^](#_ENREF_175) |
|  | ROS | *U4* | Cervical cancer | - | Inhibited by ROS | Cell culture | [^295^](#_ENREF_295) |
| PiRNA | ROS | *PiR-31470* | Prostatic cancer | PIWIL4-GSTP1 | Up | Cell culture | [^173^](#_ENREF_173) |
